# Supplementary material for: Evidence for spin-dependent energy transport in a superconductor
Source: Nat Commun. 2020 Aug 28;11:4336. doi: 10.1038/s41467-020-18161-w (PMC7455713; doi:10.1038/s41467-020-18161-w)
Supplement: Supplementary file 1 — Supplementary Information [file 41467_2020_18161_MOESM1_ESM.pdf]

Supplementary information for ”Evidence for spin-dependent energy transport in a superconductor”

M. Kuzmanović[1], B. Y. Wu[1,2], M. Weideneder[1,3], C. H. L. Quay[1], and M. Aprili[1]

[1] *Laboratoire de Physique des Solides (CNRS UMR 8502), Université Paris-Saclay, 91405 Orsay, France*
[2] *Graduate Institute of Applied Physics, National Taiwan University, Taipei 10617, Taiwan*
[3] *Institute for Experimental and Applied Physics, University of Regensburg, 93053 Regensburg, Germany*

July 21, 2020

1 Supplementary Methods

1.1 Supplementary theory

The many-body approach to BCS superconductivity is to construct the equations of motion for the Green's function g, supplemented by the equations of motion for the anomalous part, which describes the superconducting correlations [1, 2, 3, 4, 5].

In a disordered superconductor, in which the (impurity) scattering time is shorter than any other timescale in the system, one can integrate over momentum space of the Eilenberger equation [6] and obtain the Usadel equation [7], which describes the system in terms of a position and energy dependent diffusion equation.

The out of equilibrium state of the system can be described within the Keldysh formalism, in which the Green's function (GF) can be written down as

$$\check{g} = \begin{pmatrix} \hat{g}^{\mathrm{R}} & \hat{g}^{\mathrm{K}} \\ 0 & \hat{g}^{\mathrm{A}} \end{pmatrix} \tag{1}$$

where $\hat{g}^{R/A}$ is the retarded/advanced GF (related by $\hat{g}^{\rm A} = -\tau_3 \hat{g}^{\rm R} \tau_3$), and $\hat{g}^{\rm K}$ the Keldysh component which takes into account the (out of equilibrium) distribution function.

For the case of a superconductor in a spin-splitting field the Usadel equation for the GF $\check{g}$ reads:

$$D\nabla \cdot (\check{g}\nabla\check{g}) + [iE\tau_3 - i\boldsymbol{h}\cdot\boldsymbol{\sigma}\tau_3 - \check{\Delta} - \check{\Sigma}, \check{g}] = 0 \tag{2}$$

where D is the normal state diffusion coefficient, E the energy, $\boldsymbol{h}$ the external magnetic field, σ_i and τ_i the Pauli matrices in the spin and Nambu (electron-hole) subspaces respectively [1], $\check{\Delta}$ a matrix related to the (complex) order parameter, and $\check{\Sigma}$ the self energy term.

In addition to the Usadel equation 2, one also needs to consider the so-called normalizing condition $\check{g}^2 = \check{I}$, where $\check{I}$ is the unit matrix in the Nambu-spin space.

This chapter provides a detailed overview of the theory of spin-dependent transport phenomena in Zeeman-split superconductors introduced in [8], and described in more detail in [9, 10], with a focus on the spin-energy mode, first introduced in [11], and its experimental signatures. The scope of the discussion is restricted to the aspects relevant to the experiment.

1.1.1 Spectral equations

In the case of a uniform superconductor ($\hat{\Delta} = const$), in the R subspace (the "$\hat{g}^{\rm R}$" block of equation 2) the gradient term vanishes from the Usadel equation and one is left with:

$$[iE\tau_3 - i\boldsymbol{h}\cdot\boldsymbol{\sigma}\tau_3 - \hat{\Delta} - \hat{\Sigma}, \hat{g}^{\rm R}] = 0 \tag{3}$$

In the absence of gradients one can choose a gauge in which Δ is a real number, so that $\hat{\Delta}$ becomes $\hat{\Delta} = \Delta\tau_1$. In the following the self-energy contribution $\hat{\Sigma}$ describes the effects of spin relaxation through spin-flip and spin-orbit mechanisms, as well the effects of orbital depairing. Within the first

[1] formally speaking $\hat{\sigma}_i = \hat{I} \oplus \sigma_i$ and $\hat{\tau}_i = \sigma_i \oplus \hat{I}$ where σ_i is the regular definition of the Pauli matrices. For example $\tau_2 = \begin{pmatrix} 0 & 0 & -i & 0 \\ 0 & 0 & 0 & -i \\ i & 0 & 0 & 0 \\ 0 & i & 0 & 0 \end{pmatrix}$, and $\sigma_3 = \begin{pmatrix} 1 & 0 & 0 & 0 \\ 0 & -1 & 0 & 0 \\ 0 & 0 & 1 & 0 \\ 0 & 0 & 0 & -1 \end{pmatrix}$. For the σ matrices in the Usadel equation the direct product with the unity matrix is in the Nambu space is implied.

Born approximation the self-energy contributions are:

$$\begin{aligned}\check{\Sigma}_{\mathrm{SF}} &= \frac{\boldsymbol{\sigma}\cdot\tau_3\check{g}\tau_3\boldsymbol{\sigma}}{8\tau_{\mathrm{SF}}},\\ \check{\Sigma}_{\mathrm{SO}} &= \frac{\boldsymbol{\sigma}\cdot\check{g}\boldsymbol{\sigma}}{8\tau_{\mathrm{SO}}},\\ \check{\Sigma}_{\mathrm{ORB}} &= \frac{\tau_3\check{g}\tau_3}{6\tau_{\mathrm{ORB}}}\end{aligned} \tag{4}$$

In the principle the self-energy should also contain terms describing the electron-electron and electron-phonon interactions. However these terms would be non-local in energy and would increase the complexity of the problem significantly.

If the out of equilibrium state is probed within a time shorter than the effective electron-electron interaction time the effects of the pseudo-thermalization (i.e. redistribution of the quasiparticles towards a Fermi-Dirac-like distribution through particle collisions) will be small, and thus this term can be dropped. Likewise, if there is a faster relaxation mechanism than the quasiparticle recombination, which in this case is an absorbing boundary condition at the (geometrical) end of the system, the effects of the electron-phonon interaction can also be neglected. As a model without these contributions successfully captures the main physical effects observed in the experiment the omission of these terms is justified.

The normalization condition in the R subspace gives $(\hat{g}^{\mathrm{R}})^2 = 1$, which allows for a parametrization in the form $\hat{g}^{\mathrm{R}} = \sum_{j=0}^{3}\left(g^{\mathrm{R}}_{j,1}\tau_1 + g^{\mathrm{R}}_{j,3}\tau_3\right)\sigma_j$. The components proportional to τ_1 are related to the anomalous part of the GF, and the ones proportional to τ_3 are related to the regular part. Similarly the components proportional to σ_0 describe singlet correlations, while the ones proportional to $\sigma_{i=\{1,2,3\}}$ describe triplet correlations in x, y and z directions respectively. For a BCS superconductor, at zero field, we have $g^{\mathrm{R}}_{0,3} = \frac{E}{\sqrt{E^2-\Delta^2}}$ and $g^{\mathrm{R}}_{0,1} = \frac{i\Delta}{\sqrt{E^2-\Delta^2}}$, while the other ones are equal to zero. If the external magnetic field is applied along the z direction only the σ_0 and σ_3 terms need to be kept.

With the $\hat{\Delta}$ and the $\hat{\Sigma}$ as defined in eq. 4 the Usadel equation reduces to a system of nonlinear coupled algebraic equations, in terms of four complex numbers: $g_{0,1}$, $g_{3,1}$, $g_{0,3}$ and $g_{3,3}$. A numerical solution, obtained using a variant of the Powell method [12], of the Usadel equation presented in terms of the components of $\hat{g}^{\mathrm{R}}$, calculated for the following parameters: $H = 1\mathrm{T}$, $\Delta = 235\mu\mathrm{eV}$, $H = 1\mathrm{T}$, $\tau_{\mathrm{ORB}}^{-1} = 6.5\mu\mathrm{eV}$, $\tau_{\mathrm{SO}}^{-1} = 13\mu\mathrm{eV}$, $\tau_{\mathrm{SF}}^{-1} = 0$ (these are the same values as for the rest of the theoretical figures in this document, unless otherwise stated), is shown in figure 1.

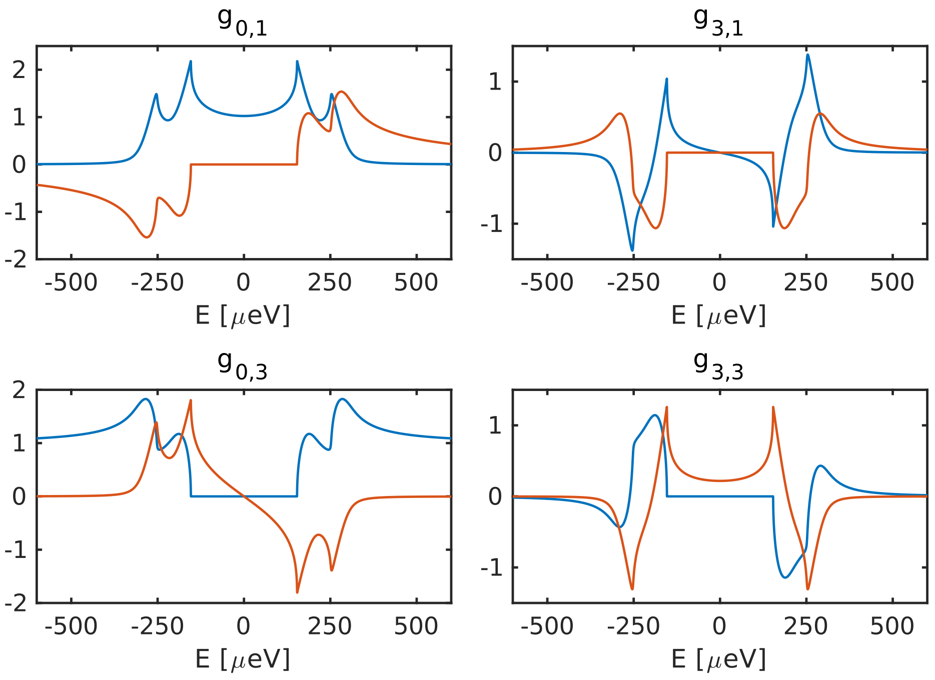

Supplementary Figure 1: A numerical solution of the Usadel equation shown in terms of the real (blue lines) and imaginary (red lines) parts of $g_{0,1}$, $g_{3,1}$, $g_{0,3}$ and $g_{3,3}$.

The spin-averaged density of states $N_+ = \frac{N_\uparrow + N_\downarrow}{2}$ is directly given by $N_+ = Re(g_{0,3})$, while the DOS spin-difference $N_- = \frac{N_\uparrow - N_\downarrow}{2}$ is given by $N_- = Re(g_{3,3})$ (see figure 1).

1.1.2 Kinetic equations

After solving the spectral equations, and obtaining $\hat{g}^{\mathrm{R}}$, we can now turn out attention to the the kinetic part of the problem, which determines the out-of-equilibrium distribution function.

By utilizing the normalization condition, $\check{g}^2 = 1$, the Keldysh component can be rewritten as $\hat{g}^{\mathrm{K}} = \hat{g}^{\mathrm{R}} \hat{f} - \hat{f} \hat{g}^{\mathrm{A}}$, where $\hat{f}$ is the generalized distribution function $\hat{f} = f_L + f_T \tau_3 + \sum_{i=1}^{3} (f_{Ti} \sigma_i + f_{Li} \sigma_i \tau_3)$. Following the same reasoning as before, if the external magnetic field is applied along the z direction, the distribution function can be reduced to:

$$\hat{f} = f_L + f_T \tau_3 + f_{\mathrm{T}3} \sigma_3 + f_{\mathrm{L}3} \sigma_3 \tau_3 \qquad (5)$$

In equilibrium only the f_L component is nonzero: $f_L^0 = \tanh(\frac{E}{2k_B T})$, which

is related to the particle distribution function in the following way $f^p(E) = (1 - f_L(E))/2$.

If we allow the distribution function to be position dependent $\hat{f} = \hat{f}(x)$, and substitute this into the Usadel equation (eq. 2), the gradient term will be nonzero. In the Keldysh subspace the equation then reads $\nabla \tilde{J} - \tilde{S} = 0$, which is in fact a continuity equation for the current $\tilde{J} = D\hat{g}^{\mathrm{R}}\nabla\hat{g}^{\mathrm{K}}$ with sink terms given by $\tilde{S} = [\check{g}, iE\tau_3 - i\boldsymbol{h}\cdot\boldsymbol{\sigma}\tau_3 - \check{\Delta} - \check{\Sigma}]^{\mathrm{K}}$.

To compute physical observables,one needs to multiply $\tilde{J}$ by an appropriate matrix and take the trace:

$$\tilde{j}_{a,b} = \frac{1}{8}\mathrm{Tr}\left[\tau_a\sigma_b\tilde{J}\right]$$

The energy current density is given by $j_e = \tilde{j}_{0,0}$ (even in the electron-hole as well as the spin subspaces), the charge current density by $j_c = \tilde{j}_{3,0}$ (odd in the e-h subspace but even in the spin subspace), the spin current density by $j_s = \tilde{j}_{0,3}$ (e-h even, spin odd), and finally the spin-energy current is given by $j_{\mathrm{se}} = \tilde{j}_{3,3}$ (odd in both subspaces). The total current is obtained by integrating these current densities with respect to energy: $J_e = \int_{-\infty}^{\infty} dE E j_{\mathrm{e}}(E)$, $J_c = e\int_{-\infty}^{\infty} dE j_{\mathrm{c}}(E)$, $J_s = \int_{-\infty}^{\infty} dE j_{\mathrm{s}}(E)$, $J_{\mathrm{se}} = \int_{-\infty}^{\infty} dE E j_{\mathrm{se}}(E)$.

If we go back to the energy resolved quantities and utilize the previously laid out parametrization for $\hat{g}^{\mathrm{R}}$ and $\hat{f}^{\mathrm{K}}$ we can obtain[2] a simple system of equations for the currents:

$$\begin{aligned} j_e &= D_{\mathrm{L}}\nabla f_{\mathrm{L}} + D_{\mathrm{T3}}\nabla f_{\mathrm{T3}}, \\ j_s &= D_{\mathrm{L}}\nabla f_{\mathrm{T3}} + D_{\mathrm{T3}}\nabla f_{\mathrm{L}}, \\ j_c &= D_{\mathrm{T}}\nabla f_{\mathrm{T}} + D_{\mathrm{L3}}\nabla f_{\mathrm{L3}}, \\ j_{\mathrm{se}} &= D_{\mathrm{T}}\nabla f_{\mathrm{L3}} + D_{\mathrm{L3}}\nabla f_{\mathrm{T}} \end{aligned} \tag{6}$$

In the equation 6 $D_{L,T,L3,T3}$ are the energy dependent diffusion coefficients

[2]As en example the energy current $j_e = \frac{1}{8}Tr\left[\tilde{J}\right]$ is found to be $j_e = \frac{D}{2}\left(1 + |g_{0,3}|^2 + |g_{3,3}|^2 - |g_{0,1}|^2 - |g_{3,1}|^2\right)\nabla f_{\mathrm{L}} + \frac{D}{2}\left(g_{3,3}g_{0,3}^* + g_{0,3}g_{3,3}^* - g_{3,1}g_{0,1}^* - g_{0,1}g_{3,1}^*\right)\nabla f_{\mathrm{T3}}$. The prefactor in front of ∇f_{L} is identified as D_L and the one in front of ∇f_{T3} as D_{T3}. The same quantities will appear in the expression for the spin current j_s. Likewise in the j_c-j_{se} subspace.

given by the equation 7 and shown in figure 2.

$$
\begin{aligned}
D_{\mathrm{L}} &= \frac{D}{2}\left(1+|g_{0,3}|^2+|g_{3,3}|^2-|g_{0,1}|^2-|g_{3,1}|^2\right),\\
D_{\mathrm{T3}} &= \frac{D}{2}\left(g_{3,3}g_{0,3}^*+g_{0,3}g_{3,3}^*-g_{3,1}g_{0,1}^*-g_{0,1}g_{3,1}^*\right),\\
D_{\mathrm{T}} &= \frac{D}{2}\left(1+|g_{0,3}|^2+|g_{3,3}|^2+|g_{0,1}|^2+|g_{3,1}|^2\right),\\
D_{\mathrm{L3}} &= \frac{D}{2}\left(g_{3,3}g_{0,3}^*+g_{0,3}g_{3,3}^*+g_{3,1}g_{0,1}^*+g_{0,1}g_{3,1}^*\right)
\end{aligned}
\tag{7}
$$

For each of these currents the sink terms can be computed in exactly the same way:

$$\tilde{s}_{a,b} = \frac{1}{8}\mathrm{Tr}\left[\tau_a\sigma_b\tilde{S}\right]$$

The continuity equation can then be restated as:

$$
\begin{aligned}
\nabla j_e &= 0,\\
\nabla j_s &= S_{\mathrm{T3}}f_{\mathrm{T3}},\\
\nabla j_c &= R_{\mathrm{T}}f_{\mathrm{T}}+R_{\mathrm{L3}}f_{\mathrm{L3}},\\
\nabla j_{\mathrm{se}} &= (R_{\mathrm{T}}+S_{\mathrm{L3}})f_{\mathrm{L3}}+R_{\mathrm{L3}}f_{\mathrm{T}}
\end{aligned}
\tag{8}
$$

The relaxation rates R_T and R_{L3} correspond to Andreev-like charge relaxation processes, while the S_{T3} and S_{L3} correspond to spin relaxation processes. One can verify, by taking the appropriate traces and separating out the terms proportional to each of the distribution function modes, that they are as follows:

$$
\begin{aligned}
R_T &= 2\Delta Re(g_{0,1}),\\
R_{\mathrm{L3}} &= 2\Delta Re(g_{3,1}),\\
S_{\mathrm{L3}} &= R_{\mathrm{S}}\left[Re(g_{0,3})^2-Re(g_{3,3})^2+\beta\left(Re(g_{3,1})^2-Re(g_{0,1})^2\right)\right],\\
S_{\mathrm{T3}} &= R_{\mathrm{S}}\left[Re(g_{0,3})^2-Re(g_{3,3})^2-\beta\left(Im(g_{3,1})^2-Im(g_{0,1})^2\right)\right]
\end{aligned}
\tag{9}
$$

where $R_{\mathrm{S}} = R_{\mathrm{SO}} + R_{\mathrm{SF}} = \frac{1}{\tau_{\mathrm{SO}}} + \frac{1}{\tau_{\mathrm{SF}}}$ and $\beta = \dfrac{R_{\mathrm{SF}} - R_{S0}}{R_S}$. Supplementary figure 2 also shows the relaxation rates, calculated for the same parameters as figure 1.

At $H = 0$ all of the $g_{3,i}$ components vanish and D_{T3} and D_{L3} do so too.

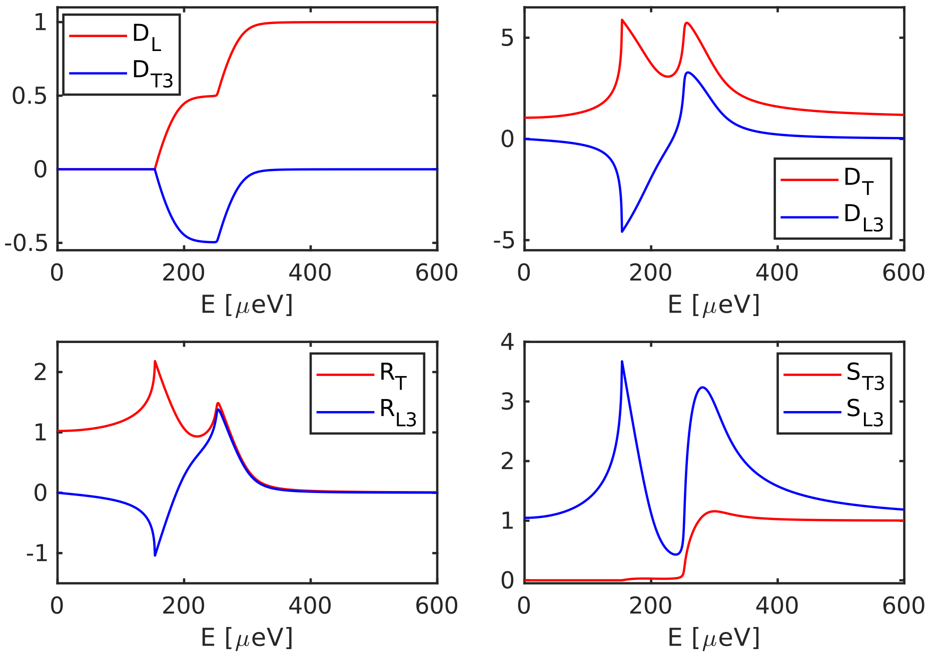

Supplementary Figure 2: Top: the normalized ($D = 1$) energy dependent diffusion coefficients D_L and D_{T3} (left) as well as D_T and D_{L3} (right). Bottom: the charge (R_T and R_{L3}) and spin (S_{L3} and S_{T3}) relaxation rates, normalized to $2\Delta = 1$ and $R_{\mathrm{S}} = 1$;

The expressions for the currents 6 are reduced to:

$$\begin{aligned} j_e &= D_{\mathrm{L}} \nabla f_{\mathrm{L}}, \\ j_s &= D_{\mathrm{L}} \nabla f_{\mathrm{T3}}, \\ j_c &= D_{\mathrm{T}} \nabla f_{\mathrm{T}}, \\ j_{\mathrm{se}} &= D_{\mathrm{T}} \nabla f_{\mathrm{L3}} \end{aligned} \tag{10}$$

The transport of the spin dependent modes f_{T3} and f_{L3} is then decoupled and the transport equations 10 recover the result from [11] (without the supercurrent contribution), giving a more direct interpretation of the spin-energy current as the spin resolved energy current.

1.1.3 Transport eigenmodes

The transport problem for the four out-of-equilibrium modes can be separated into two independent subspaces: the first two equations from each of the systems 6-9 are expressed only in terms of the f_L and f_{T3} modes, while the remaining two are dependent only on f_T and f_{L3}. In the following two

subsections the transport equations in these two subspaces will be addressed separately.

The f_L and f_{T3} modes:

The $f_L - f_{T3}$ subspace of equation 6 can be rewritten as

$$\begin{pmatrix} j_e \\ j_s \end{pmatrix} = \begin{pmatrix} D_L & D_{T3} \\ D_{T3} & D_L \end{pmatrix} \nabla \begin{pmatrix} f_L \\ f_{T3} \end{pmatrix} \tag{11}$$

while the same subspace of equation 9 reads

$$\nabla \begin{pmatrix} j_e \\ j_s \end{pmatrix} = \begin{pmatrix} 0 \\ S_{T3} f_{T3} \end{pmatrix} \tag{12}$$

Substituting the first one into the second yields

$$\begin{pmatrix} D_L & D_{T3} \\ D_{T3} & D_L \end{pmatrix} \nabla^2 \begin{pmatrix} f_L \\ f_{T3} \end{pmatrix} = \begin{pmatrix} 0 \\ S_{T3} f_{T3} \end{pmatrix} \tag{13}$$

With the ansatz $\begin{pmatrix} f_L \\ f_{T3} \end{pmatrix} = e^{kx}\mathbf{v}$, we have

$$\begin{pmatrix} k^2 D_L & k^2 D_{T3} \\ k^2 D_{T3} & k^2 D_L - S_{T3} \end{pmatrix} \mathbf{v} = 0 \tag{14}$$

which has a nontrivial solution only if the determinant of the matrix is zero. Solving for k yields two solutions $k_1^2 = 0$ and $k_2^2 = \frac{D_L S_{T3}}{D_L^2 - D_{T3}^2}$, with the corresponding null-space vectors $\mathbf{v}_1 \propto \begin{pmatrix} 1 \\ 0 \end{pmatrix}$ and $\mathbf{v}_2 \propto \begin{pmatrix} -D_{T3} \\ D_L \end{pmatrix}$.

The top panel of figure 3 shows the energy dependence of k_2.

The first solution corresponds to $\nabla^2 f_L = 0$ because of the absence of any inelastic relaxation mechanism in the model. The effective relaxation is taken into account through an absorbing boundary condition at the ends of the wire (where large, well thermalized, metallic reservoirs are located). Assuming that the quasiparticles are injected at $x = 0$ and the reservoir is located at $x = L$ the solution can be expressed as:

$$\begin{pmatrix} f_L \\ f_{T3} \end{pmatrix} = B_1 \mathbf{v}_1 (L - x) + B_2 \mathbf{v}_2 e^{-k_2 x} + B_2^+ \mathbf{v}_2 e^{+k_2 x} + \begin{pmatrix} f_L^0 \\ 0 \end{pmatrix}$$

Once again, invoking the boundary condition $\begin{pmatrix} f_L \\ f_{T3} \end{pmatrix}_{x=L} = \begin{pmatrix} f_L^0 \\ 0 \end{pmatrix}$, where $f_L^0 = \tanh(\frac{E}{2k_B T})$, one finds $B_2^+ = -B_2 e^{-2k_2 L}$

Finally the full form of the solution is found to be:

$$\begin{pmatrix} f_L \\ f_{\mathrm{T3}} \end{pmatrix} = B_1 \begin{pmatrix} L - x \\ 0 \end{pmatrix} + B_2 \mathbf{v}_2 \big(e^{-k_2 x} - e^{+k_2 (x - 2L)} \big) + \begin{pmatrix} f_L^0 \\ 0 \end{pmatrix} \tag{15}$$

The energy dependent coefficients B_1 and B_2 are to be determined from the boundary condition at the point of quasiparticle injection. This is discussed in detail in section 1.1.3.

At vanishing magnetic field the transport eigenmodes in the f_L-f_{T3} subspace decouple as well. The second mode describes just the decay of the spin mode, which then relaxes within the spin-flip length (S_{L3} is nonzero even at $H = 0$). The transport of the f_L mode is the same as in [13], only modified by the energy dependent diffusion D_L.

The f_T and f_{L3} modes:

By following the same procedure as in the previous section one can obtain the transport eigenmodes in the $f_T - f_{\mathrm{L3}}$ subspace. From the equations 6 and 8 we have:

$$\begin{pmatrix} k^2 D_T - R_T & k^2 D_{\mathrm{L3}} - R_{\mathrm{L3}} \\ k^2 D_{\mathrm{L3}} - R_{\mathrm{L3}} & k^2 D_{\mathrm{T}} - R_T - S_{\mathrm{L3}} \end{pmatrix} \mathbf{v} = 0 \tag{16}$$

In the limit of vanishing spin relaxation ($S_{\mathrm{L3}} = 0$) two modes can be obtained with $k_3 = \dfrac{R_{\mathrm{L3}} - R_T}{D_{\mathrm{L3}} - D_T}$, $\mathbf{v}_3 \propto \begin{pmatrix} -1 \\ 1 \end{pmatrix}$ and $k_4 = \dfrac{R_{\mathrm{L3}} + R_T}{D_{\mathrm{L3}} + D_T}$, $\mathbf{v}_4 \propto \begin{pmatrix} 1 \\ 1 \end{pmatrix}$.

Taking into account the boundary condition, the following solution can be obtained:

$$\begin{pmatrix} f_T \\ f_{\mathrm{L3}} \end{pmatrix} = B_3 \mathbf{v}_3 \big(e^{-k_3 x} - e^{+k_3 (x - 2L)} \big) + B_4 \mathbf{v}_4 \big(e^{-k_4 x} - e^{+k_4 (x - 2L)} \big) \tag{17}$$

If the spin relaxation rate is finite the form of the solution 17 remains the same, however $\mathbf{v}_3$ and $\mathbf{v}_4$ become energy dependent, while the expressions for k_3 and k_4 become slightly more complicated. The bottom panel of figure 3 shows the relaxation rates for both modes based on the calculation including the spin-relaxation mechanism.

At energies close to the Δ the transport of the f_T and f_{L3} modes is closely coupled (because $D_{\mathrm{L3}} \neq 0$). However at higher energies $D_{\mathrm{L3}} \to 0$ so the modes become decoupled. We can also see this by investigating the components of the k_3 and k_4 modes in the basis of f_{T} and f_{L3}. The decomposition of $\mathbf{v}_3$ and $\mathbf{v}_4$ is shown in figure 4; indeed at higher energies the k_3 mode becomes the charge mode, while the k_4 becomes the spin-energy mode.

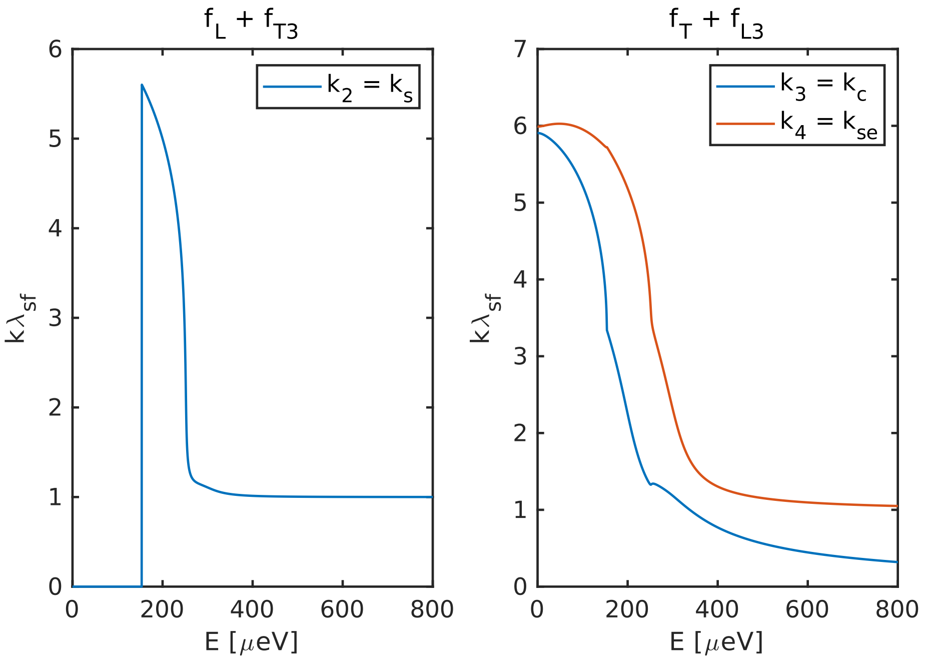

Supplementary Figure 3: The relaxation rates of the exponentially decaying modes normalized by the spin-relaxation length $\lambda_{\rm SF} = \sqrt{D\tau_{\rm SO}}$ in the $f_L - f_{\rm T3}$ (left) and $f_T - f_{\rm L3}$ (right) subspaces. The labels are assigned to the traces based on the high energy behavior, as shown in figure 4.

Unlike the previous two modes the f_T and $f_{\rm L3}$ are not completely decoupled at $H = 0$ as the relaxation (equation 9) mixes the two even at all magnetic fields.

Injector boundary conditions:

In the experiment that was performed the injector junction is an NIS tunnel junction ($Cu/Al_2O_3/Al$). The current density through this junction is given by equation 18:

$$\begin{pmatrix} j_e \\ j_s \\ j_c \\ j_{\rm se} \end{pmatrix} = \frac{1}{\tilde{R}} \begin{pmatrix} N_+ & N_- & 0 & 0 \\ N_- & N_+ & 0 & 0 \\ 0 & 0 & N_+ & N_- \\ 0 & 0 & N_- & N_+ \end{pmatrix} \begin{pmatrix} \tilde{f}_{\rm L} \\ \tilde{f}_{\rm T3} \\ \tilde{f}_{\rm T} \\ \tilde{f}_{\rm L3} \end{pmatrix} \tag{18}$$

where $\tilde{R}$ is the barrier resistance normalized by the ratio of the junction surface area and the cross section of the wire. $\tilde{f}_i = f_i - f_i^N$ is the difference between the distribution function in the superconductor and in

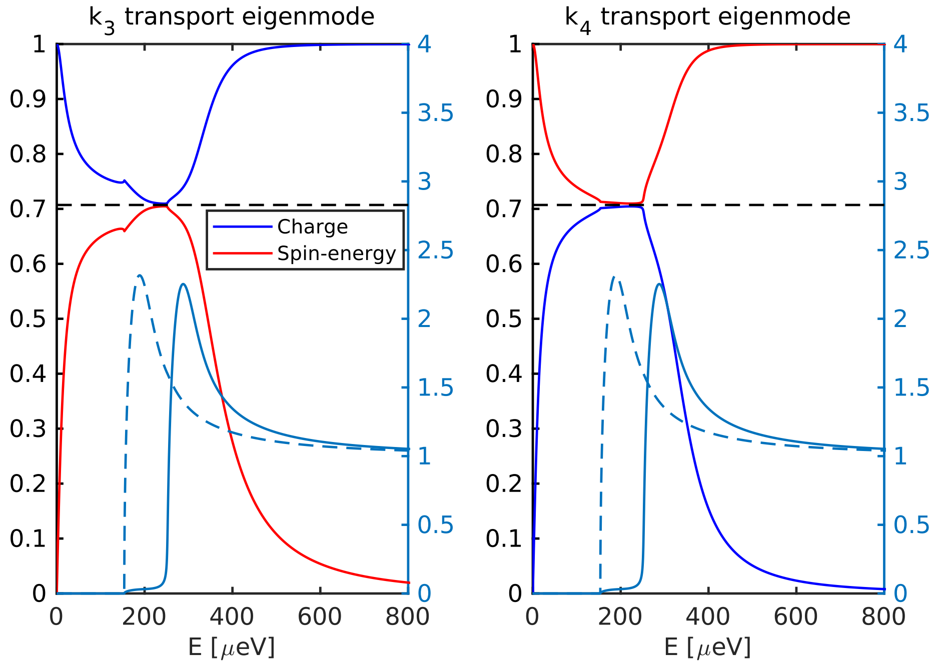

Supplementary Figure 4: The decomposition of the k_3 (left) and (k_4) transport eigenmodes in terms of f_T (charge, blue line) and f_{L3} (spin-energy, red line). The dashed black line indicates an equal mixture of the f_T and f_{L3} modes. Note that below $E \approx 150\mu\mathrm{eV}$ there are no quasiparticle states as indicated by the DOS (shown on the right scale).

the normal metal (indicated by the superscript N). The tunneling matrix is block-diagonal and again separates the problem into the $f_L - f_{\mathrm{T3}}$ and $f_T - f_{\mathrm{L3}}$ subspaces.

More generally, when using a ferromagnetic injector the off-diagonal blocks of 18 are also nonzero and are proportional to the injector polarization P (cf. equation 57 in [10]), allowing for the excitation of the spin-dependent modes without Zeeman splitting. Here the we restrict the discussion only to the case of a normal-metal injector junction.

When the superconductor is Zeeman split $N_- \neq 0$, and even if the spin (f_{T3}) and spin-energy (f_{L3}) modes are not present a finite spin and spin energy current will flow through the barrier: $j_s = \frac{N_-}{\tilde{r}} \tilde{f}_L$ and $j_{\mathrm{se}} = \frac{N_-}{\tilde{r}} \tilde{f}_T$.

This current through the tunnel barrier must be component-wise equal to the current along the wire at $x = 0$; there are two components to this current the one which flows to the left $j_\leftarrow$ (and relaxes in the reservoir at $x = -L_L < 0$) and the one which flows to the right $j_\rightarrow$ (relaxes at $x = L_R > 0$). For the sake of simplicity only the case of a symmetric wire ($L_L = L_R = L$)

will be discussed here, under which $|j_{\leftarrow}| = |j_{\rightarrow}|$ and allows one to consider only the right-moving current with an effective barrier resistance $\tilde{r} = 2\tilde{R}$.

In the $f_L - f_{\mathrm{T3}}$ subspace the current conservation is given by (from equations 18 and 13):

$$\begin{pmatrix} D_{\mathrm{L}} & D_{\mathrm{T3}} \\ D_{\mathrm{T3}} & D_{\mathrm{L}} \end{pmatrix} \nabla \begin{pmatrix} f_L \\ f_{\mathrm{T3}} \end{pmatrix}_{x=0} - \frac{1}{\tilde{r}} \begin{pmatrix} N_+ & N_- \\ N_- & N_+ \end{pmatrix} \begin{pmatrix} f_{\mathrm{L}} \\ f_{\mathrm{T3}} \end{pmatrix}_{x=0} = \frac{1}{\tilde{r}} \begin{pmatrix} N_+ & N_- \\ N_- & N_+ \end{pmatrix} \begin{pmatrix} f_{\mathrm{L}}^N \\ f_{\mathrm{T3}}^N \end{pmatrix} \tag{19}$$

When a voltage is applied to the normal metal the distribution function is shifted by the voltage $f(E,V) = f_0(E-V)$. Tunneling across the barrier will *imprint* this distribution into the superconductor, resulting in quasiparticle up to the energy $E \approx eV$, which can be parameterized by the four out of equilibrium modes. The normal metal will be driven out of equilibrium by this process, and kinetic equations should be solved for the nonequilibrium state.

Because of the large thickness of the injector electrode compared to the thickness of the Al wire the distribution function is assumed to be the Fermi-Dirac one offset by the applied voltage - for one tunneled electron the distribution function is modified by $V_N N_N \delta f_N = 1 = V_S N_S \delta f_S$ (V is the volume and N the density of states at the Fermi level, the subscript N/S refers to the normal metal or the superconductor). As $V_N \gg V_S$ the superconductor is driven further out equilibrium, for a relatively small δf_S one can safely neglect the nonequilibrium state in the normal metal.

The right hand side of 19 depends solely on the distribution function of the injection electrode; when a finite voltage is applied in the L/T parametrization the distribution function is $f_{L/T}^N = \left(\tanh \frac{E-eV}{k_B T} \pm \tanh \frac{E-eV}{k_B T}\right)/2$.

By using the expression 15 the left hand side can be expressed in terms of B_1 and B_2. By solving the linear system of equations, and making all the necessary substitutions, at the end one finds that the distribution function in the superconducting wire is given by:

$$\begin{aligned} f_L(E,x) &= \chi_{\mathrm{L}}(E,x) f_L^N(E,V,T) + f_L^0(E,T), \\ f_{\mathrm{T3}}(E,x) &= \chi_{\mathrm{T3}}(E,x) f_L^N(E,V,T), \\ f_T(E,x) &= \chi_{\mathrm{T}}(E,x) f_T^N(E,V,T), \\ f_{\mathrm{L3}}(E,x) &= \chi_{\mathrm{L3}}(E,x) f_T^N(E,V,T) \end{aligned} \tag{20}$$

The χ coefficients of equation 20, calculated for the same parameters as in figure 1 and $x = 0$, are shown in figure 5.

If $L_L \neq L_R$ the left and right moving currents are not the same, and a set of B parameters (eqs 15 and 17) determines both of them. However, by imposing the continuity of the distribution function at $x = 0$: $f_{\leftarrow}(x=0) =$

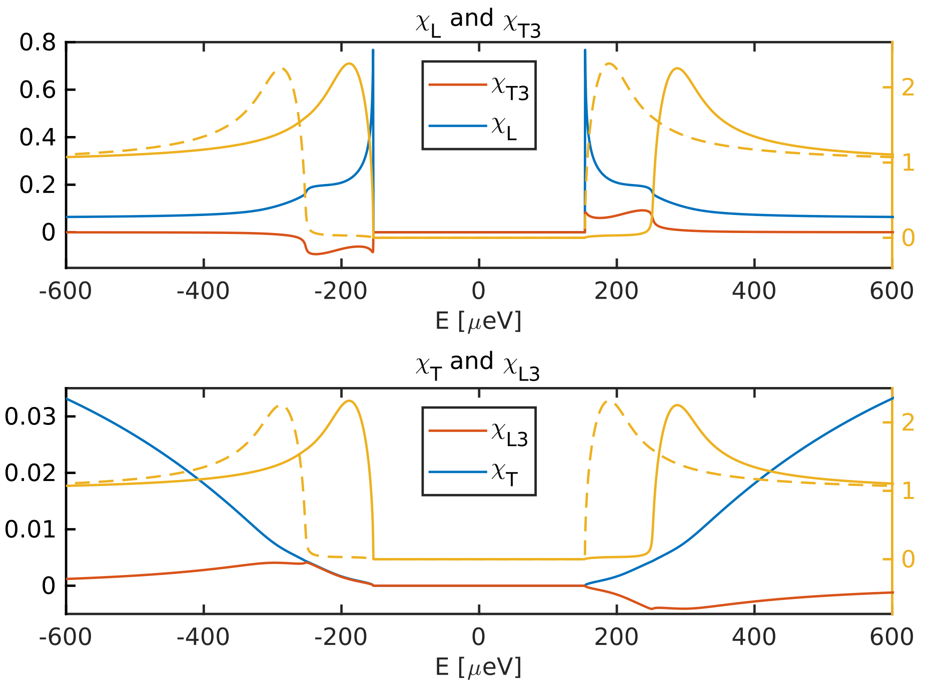

Supplementary Figure 5: The χ coefficients from equation 20 calculated for the experimental parameters at $H = 1$T and $x = 0$. The top panel shows χ_L and χ_{T3} while the bottom one shows χ_T and χ_{L3}; in both panels the DOS for both spins is shown on the right scale.

$f_{\rightarrow}(x = 0)$, the $B_{\leftarrow}$ can be expressed in terms of $B_{\rightarrow}$ and the problem is effectively reduced to the symmetric case.

In the experiment the corresponding values are $L_R \approx 6\mu$m, and $L_L \approx 4\mu$m. For the sake of simplicity, the calculations have been performed with $L_R = L_L = 5\mu$m.

1.1.4 Spin and charge accumulation

The currents introduced in section 1.1.2 have a corresponding charge (density), which is defined as

$$q_{\tilde{a,b}} = \frac{1}{8}\mathrm{Tr}\left[\tau_3\tau_a\sigma_b\hat{g}^{\mathrm{K}}\right]$$

Compared to the definition of the currents there is an extra τ_3 matrix, coming from the structure of the time derivative term in the Gor'kov equation [1].

In terms of the components of the retarded Green's function and the generalized distribution function the accumulation of charge, spin, energy

and spin-energy is respectively given by:

$$
\begin{aligned}
\mu &= \int_{-\infty}^{\infty} \mathrm{Tr}\big[g(\hat{E},x)^{\mathrm{K}}\big]dE = \int_{-\infty}^{\infty} \big[f_T\mathrm{Re}(g_{0,3}) + f_{\mathrm{L3}}\mathrm{Re}(g_{3,3})\big]dE,\\
\mu_z &= \int_{-\infty}^{\infty} \mathrm{Tr}\big[\tau_3\sigma_3 g(\hat{E},x)^{\mathrm{K}}\big]dE = \int_{-\infty}^{\infty} \big[f_{\mathrm{T3}}\mathrm{Re}(g_{0,3}) + f_{\mathrm{L}}\mathrm{Re}(g_{3,3})\big]dE,\\
\epsilon &= \int_{-\infty}^{\infty} \mathrm{Tr}\big[\tau_3 g(\hat{E},x)^{\mathrm{K}}\big]EdE = \int_{-\infty}^{\infty} \big[f_L\mathrm{Re}(g_{0,3}) + f_{\mathrm{T3}}\mathrm{Re}(g_{3,3})\big]EdE,\\
\epsilon_z &= \int_{-\infty}^{\infty} \mathrm{Tr}\big[\sigma_3 g(\hat{E},x)^{\mathrm{K}}\big]EdE = \int_{-\infty}^{\infty} \big[f_{\mathrm{L3}}\mathrm{Re}(g_{0,3}) + f_{\mathrm{T}}\mathrm{Re}(g_{3,3})\big]EdE
\end{aligned}
\tag{21}
$$

The same result can be found using a more straight forward argument: in the particle (or electron-like) definition of the distribution function, and the semiconductor definition of the DOS, the density of spin up/down particles is given by $\rho_{\uparrow/\downarrow} = N_{\uparrow/\downarrow}(E) f^p_{\uparrow/\downarrow}(E)$, where $N_{\uparrow/\downarrow}$ is the density of states for each of the spins and f^p is the particle distribution function (i.e. in equilibrium the Fermi-Dirac distribution). The total (energy-independent) density of particles can be obtained by integrating this quantity $\rho^{tot}_{\uparrow/\downarrow} = \int_{-\infty}^{\infty} dE \rho_{\uparrow/\downarrow}(E)$. The total charge is then related to the number of particles present in the system $\rho^{tot} = \rho_\uparrow + \rho_\downarrow$. To obtain the charge imbalance one just needs to subtract the number of particles in equilibrium $\rho^{tot}_{eq} = \int_{-\infty}^{\infty} N_+(E) f_0(E,T)dE$ where f_0 is the Fermi-Dirac distribution. Likewise the total magnetization can be determined as the difference between the number of spin up and spin down quasiparticles. By using the expressions for the density of states $N_{\downarrow/\uparrow} = N_+ \pm N_-$ and the distribution function $f_{\uparrow/\downarrow} = (1 - f_L - f_T \pm (f_{\mathrm{L3}} + f_{\mathrm{T3}}))/2$ and by dropping the terms that yield zero under integration the same expressions for μ and μ_z are obtained.

Finally, to determine the out-of-equilibrium component of the charge and spin accumulation the equilibrium one must subtract the equilibrium one (i.e. to replace f_L with $f_L - f_L^0$ in eq. 21):

$$
\begin{aligned}
\mu_z &= \int_{-\infty}^{\infty} dE\big[\big(f_L(E) - f_L^0(E)\big)N_- + f_{\mathrm{T3}}N_+\big],\\
\mu &= \int_{-\infty}^{\infty} dE\big[f_T(E)N_+ + f_{\mathrm{L3}}N_-\big]
\end{aligned}
\tag{22}
$$

The first equation, describing the spin accumulation, tells us that in the presence of a spin-splitting field the energy mode (f_L) results in a finite magnetization. In equilibrium and at nonzero temperatures this is the effect that results in a finite paramagnetic response of a superconductor [14]. The second equation describes the charge imbalance of the system: the first term

describes the charge mode described by [15], while the second term describes the charge accumulation associated with the spin-energy mode.

Supplementary figure 6 shows the magnetic field dependence of the spin accumulation as well as the charge accumulation proportional to the f_T and f_{L3} modes versus the injection voltage (the field dependence of $\Delta(H)$ is as discussed in the section 1.1.5). The spin accumulation is dominantly due to the induced f_L mode and therefore grows with the magnetic field (as N_- becomes larger with increasing H), and again decays when $\Delta \to 0$ as the field approaches the critical one.

As discussed in [16, 17], the orbital depairing induced by the magnetic field facilitates the charge relaxation processes and therefore the f_T mode is suppressed by a finite magnetic field. However as the charge imbalance due to the spin-energy mode μ_{L3} is a function of N_- it shows a qualitatively different behavior: although the charge relaxation increases at a finite filed, so does the span of energies at which N_- is nonzero. Combined these two contributions result in a charge imbalance which is maximal at $\Delta - \mu_B H < V_{inj} < \Delta - \mu_B H$ and doesn't monotonically decrease with the magnetic filed, as can be seen in figure 6.

1.1.5 The self-consistency relation

Within the Keldysh formalism the self-consistency for the pairing potential Δ is given by

$$\Delta = \frac{\lambda}{16i} \int_{-\omega_D}^{\omega_D} Tr\big[(\tau_1 - i\tau_2) g^{\mathrm{K}}(E)\big] dE$$

where λ is the BCS pairing potential, ω_D is the Debye frequency, and the trace essentially "separates out" the anomalous part of the Keldysh component.

This expression can be expanded in terms of the distribution functions and the various components of the retarded GF:

$$\Delta = \frac{\lambda}{2} \int_{-\omega_D}^{\omega_D} dE \big[\mathrm{Im}(g_{0,1}) f_L + \mathrm{Im}(g_{3,1}) f_{\mathrm{T3}} + i\big(\mathrm{Re}(g_{0,1}) f_T + \mathrm{Re}(g_{3,1}) f_{\mathrm{L3}}\big)\big] \tag{23}$$

The first term is nonzero even at equilibrium (with $f_L = \tanh(\frac{E}{2k_B T})$), while the other three are nonzero only out of equilibrium. The last two terms are related to the charge imbalance (given by the f_T and f_{L3}) and add a nonzero imaginary component to the Δ.

While it is true that at equilibrium one can always choose a gauge such that Δ is strictly real, out of equilibrium this is not the case. As it was shown in section 1.1.2, the quasiparticle charge current is relaxed through Andreev

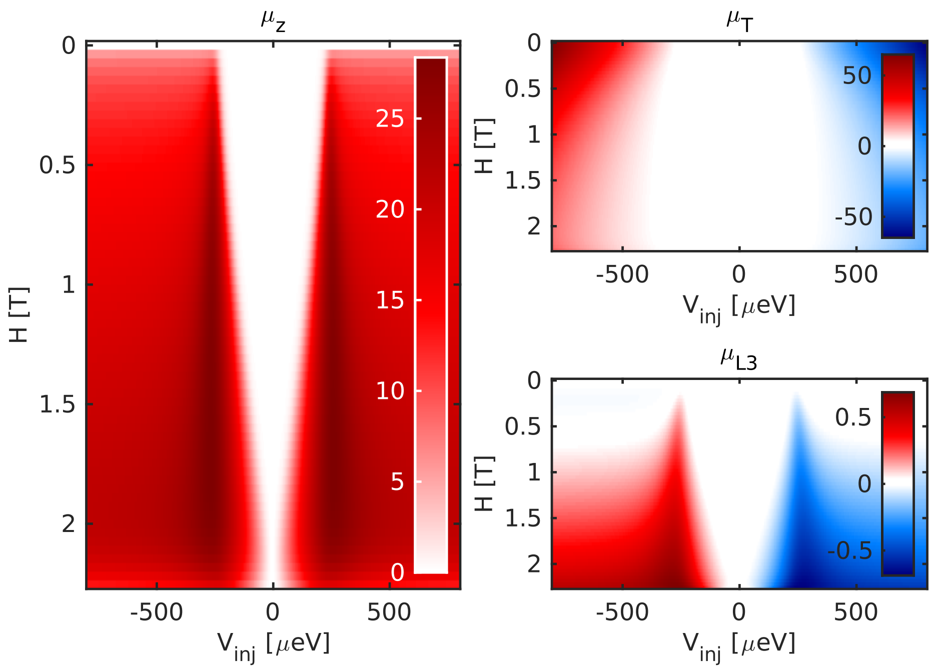

Supplementary Figure 6: A color-plot showing the spin accumulation (left panel), the charge accumulation proportional associated with the f_T (top right) and f_{L3} (bottom right) modes as a function of the applied magnetic field and the injection voltage.

processes, which means that the current is transferred from the quasiparticles to the condensate, which implies the existence of a finite phase gradient along the wire, which is precisely the meaning of the imaginary component of Δ in equation 23.

In order to solve the whole problem self-consistently one must:

- Solve the spectral equations at each position using the local $\Delta(x)$;
- Solve the transport problem in terms of all of the out-of-equilibrium modes (at each energy);
- Calculate the new $\Delta(x)$ using the self-consistency relation using the obtained modes at each x;
- Repeat the previous steps until convergence is reached.

While doing so one cannot rely on the previously laid out analytical results: the transport solution can no longer be obtained by diagonalizing the transport equations as the diffusion and relaxation terms are now position

dependent, as Δ is a function of the position as well. Therefore the solution needs to be obtained numerically from start to finish, including at the boundary condition, which increases the complexity of the calculation drastically. We can, however, calculate the $\Delta(x, V_{inj})$ based on the analytical solution (without any gradients in Δ) to estimate the magnitude of the effect in our experimental situation. Supplementary figure 7 shows the absolute value, as well as the argument of Δ at $x = 0$ (where one can naively expect the largest deviation from the equilibrium values) - the maximum suppression turns out to be in the order of $\approx 10\%$, justifying the previous approach.

The self-consistency relation in equilibrium as function of the magnetic field will be further discussed in the section describing the sample properties (1.2 and 1.2.1).

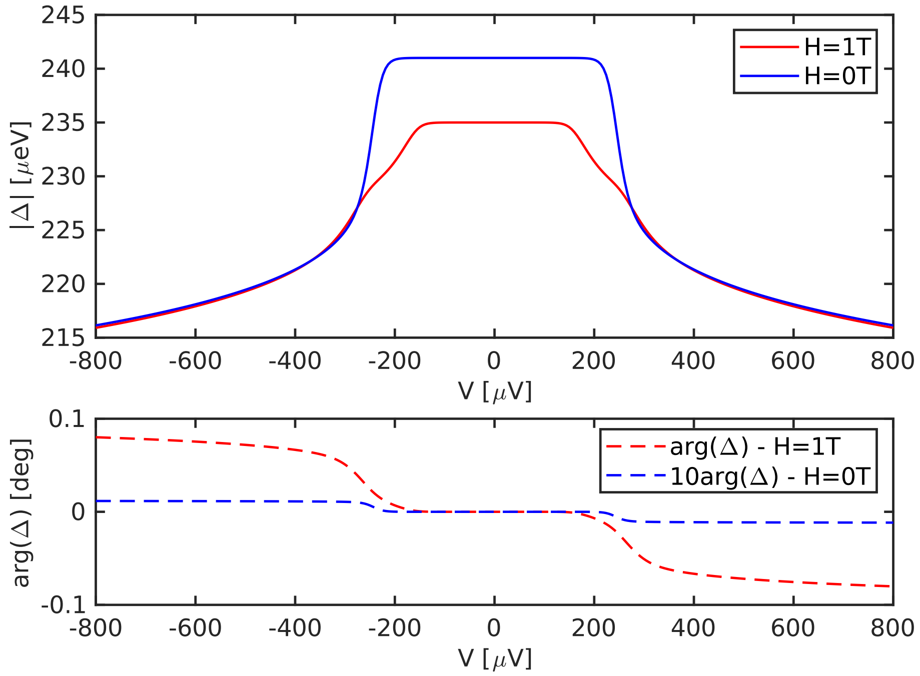

Supplementary Figure 7: The self-consistent calculation of Δ at the injection sight ($x = 0$) as a function of the injection voltage at $H = 0$T (blue) and $H = 1$T (red). The top panel shows the absolute value of the complex Δ, while the bottom shows the argument. At $H = 0$ the argument is multiplied by a factor of 10 for clarity - this signifies that the supercurrent induced in the wire is lower at $H = 0$, as it should be for a slower charge relaxation.

1.2 Supplementary experimental methods

The sample, shown in figure 8, consists of a $L = 10\mu$m long, $w = 200$nm wide superconducting Al wire. The total thickness of the wire, including the natively grown oxide layer on the top of the wire, is $d \approx 6$nm. The wire resistance at $T = 4$K is $R \approx 850\Omega$ resulting in a resistance per square of $R_\square \approx 17\Omega$ and the normal state diffusion coefficient of $D \approx 11\frac{\text{cm}^2}{\text{s}}$. The critical temperature of the wire is $T_c \approx 1.7$K (the increase of the critical temperature compared to the bulk value of ≈ 1.2K is related to the disorder induced by the small film thickness and is consistent with previous findings [18]), while the critical in plane magnetic field is $H_c \approx 2.7$T. On both ends the wire is terminated with large, well thermalized, metallic reservoirs.

On top of the wire there are several tunnel junctions (using the native oxide as the tunnel barrier):

- The injector junction J_{inj} (cyan in 8) - an NIS junction used for creating quasiparticle excitations in the wire by current injection. The normal metal N is Cu (100nm thick), the surface area of the junction is $S = 200\text{nm} \times 200\text{nm}$ and the normal state resistance is $R = 13\text{k}\Omega$.

- The detector junctions $J_{\{1,2,3\}}$ (red in 8) - SIS′ junctions, where the counter electrode S′ is an $d_{Al} \approx 8$nm thick layer of Al with a mono-layer of Pt ($d_{Pt} = 1$Å nominally) on top. The purpose, and the effects, of the Pt layer are described in detail in 1.2.3. The surface area of these junctions is $S = 50\text{nm} \times 200\text{nm}$, while their normal state resistances, as well as their distances from the injector junction are:

  - J_1: $R = 31.2\text{k}\Omega$, $L_1 = 250$nm
  - J_2: $R = 38.3\text{k}\Omega$, $L_2 = 1.89\mu$m
  - J_3: $R = 29.5\text{k}\Omega$, $L_2 = 3.53\mu$m

The basic idea of the experiment is the following: quasiparticles are injected into the wire by applying a current through J_{inj}. As the diffusion time to the reservoirs $\tau_{dif} = \dfrac{L_{res}^2}{D} \approx 20$ns is much shorter than the quasiparticle-quasiparticle recombination time $\tau_{rec} \approx 400$ns [19] the quasiparticles relax only by diffusing to the end of the wire and thermalizing with the phonon bath there. An externally applied magnetic field will cause pair-breaking effects, and for an in-plane field the pair breaking energy is determined to be $\alpha \approx 6.5\frac{\mu eV}{T^2}$ (see figures 9 and 10). Consequently, all the way up to the critical field $\alpha H_c^2 < \mu_B H$, which implies that the DOS of the superconductor

will be Zeeman split, and allow us to create spin-polarized excitations by biasing the injector junction such that $\Delta - \mu_B H < V_{inj} < \Delta - \mu_B H$.

The out-of-equilibrium quasiparticles can then be probed by one of three spin-sensitive spectroscopic detectors, positioned at different distances away from the injection site. The spin relaxation mechanism in the wire is assumed to be through spin-orbit scattering and the effective relaxation time is estimated, based on [20], to $\tau_{\mathrm{SO}} = 50\mathrm{ps}$, giving a spin relaxation length of $L_{\mathrm{SO}} \approx 240\mathrm{nm}$, which is comparable to the distance between J_{inj} and J_1. Thus our device, due to the spin sensitivity of the detector, allows for the detection of a spin dependent distribution function at short distances from the injection site.

All of the measurements presented in this chapter were performed in a ^{3}He/^{4}He dilution refrigerator at $T = 90\mathrm{mK}$.

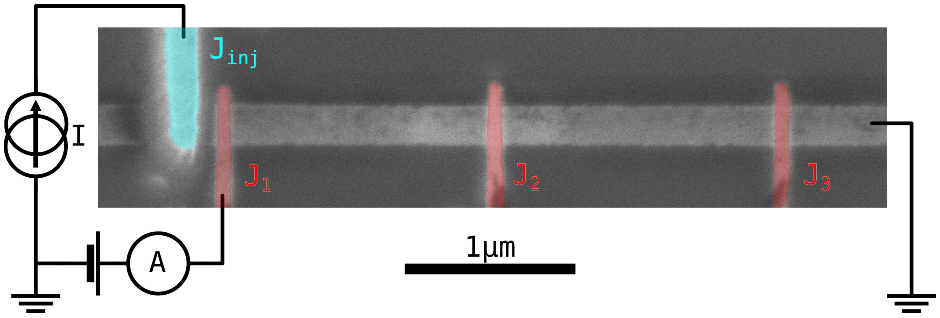

Supplementary Figure 8: An SEM micrograph of the sample, with a simplified schematic of the principal measurement setup: J_{inj} (cyan) is current biased, and the $I(V)/G(V)$ curve of one of the detector junctions $J_{\{1,2,3\}}$ (red) is measured simultaneously.

The following sections will address the injection scheme (section 1.2.1) as well as the workings of the detectors in detail (sections 1.2.2 and 1.2.3).

1.2.1 The NIS injector

In a superconductor quasiparticles can be excited by current (or voltage) biasing an NIS junction, where the tunnel barrier allows for a finite voltage drop across the junction and thus quasiparticles with energies up to the voltage bias, $E \approx eV$, can be injected into the superconductor. Following [21] the tunneling current through such a junction is given by:

$$I(V) = \frac{1}{eR_{\mathrm{N}}} \int N(E)[f^p(E) - f_N^p(E - eV)]dE \qquad (24)$$

while the differential conductance is:

$$G(V) = \frac{\partial I(V)}{\partial V} = \frac{1}{eR_{\mathrm{N}}} \int N(E) \frac{\partial f_N^p(E - eV)}{\partial V} dE \tag{25}$$

The quantity $\frac{\partial f_N^p(E - eV)}{\partial V}$ becomes the Dirac-delta function at $T = 0$, and at finite temperatures is a bell-like curve with a FWHM of $\approx 3.5k_BT$. Therefore the $G(V)$ curve of the NIS junction depends only on the DOS of the superconductor and the effective temperature in the normal metal (and at a sufficiently low temperature the $G(V)$ approaches the DOS of the superconductor).

When a finite magnetic field is applied to a superconductor there are several observable effects [22]. The first of which is the orbital (Abrikosov-Gor'kov) depairing, from the induced screening supercurrent, which results in a rounding of the DOS coherence peaks as well as a reduction of the spectroscopic gap below Δ. The strength of this effect is geometry dependent, and for a thin film superconductor with an in-plane field the depairing parameter is $\alpha = \frac{De^2d^2}{6\hbar}H^2$ [21, 23], where d is the sample thickness and D the normal state diffusion constant. The critical field, at zero temperature, due to the orbital depairing is set by $2\alpha(H) = \Delta(H = 0)$, and at low fields the $\Delta(H)$ curve is roughly linear: $\Delta(H) \approx \Delta_0 - 0.4\alpha(H)$. If the sample is thin α is quadratically suppressed which leads to an increased H_c. The second effect is the Zeeman splitting of the DOS, a result of the coupling of the quasiparticle spin degree of freedom with the external field, which shifts the spin up/down quasiparticle dos by $E_z = \pm\mu_BH$ (the Landé factor is $g = 2$) [24]. The Zeeman splitting can be observed only if the orbital smearing of the DOS is sufficiently small and if the (critical) field is larger than the temperature of the superconductor $\mu_BH > 3.5k_BT$. In the presence of the spin-orbit coupling, which is the relevant spin relaxation mechanism for this experiment, spin is only approximately a good quantum number, which leads to the spin-mixing of the DOS, as shown in figure 4 and [25] - the spin up DOS is nonzero even in the $\Delta - \mu_BH < E < \Delta + \mu_B$. However this is not directly observable with an NIS junction with a spin-independent transmission.

Supplementary figure 9 shows the $G(V)$ of the injector at $H = 0$T and at $H = 1$T. Both of these traces show features beyond the simple equilibrium model. In particular, at $H = 0$T (fig. 9, the blue trace) the coherence peaks are sharper (higher and narrower) than what one should expect at $T = 90$mK.

This can be understood in terms of the out-of-equilibrium suppression of Δ: when a finite voltage is applied across the junction there is a nonzero

current flowing through it which, due to the relatively low resistance of the junction $R(J_{inj}) = 13\text{k}\Omega$ and the thin superconducting wire, induces an out of equilibrium state in the wire, which could be taken into account through the voltage dependent gap $\Delta = \Delta(V_{inj})$. This suppression of Δ reduces the range of injection voltages for which $eV_inj \approx \Delta(V_{inj})$ and thus makes the coherence peak sharper than at equilibrium.

At $H = 1$T (fig. 9, the red trace), there are two observable peaks, corresponding to the spin down and spin up component of the DOS. The spin down peak, located at $V \approx 175\mu$V is slightly sharper than the model, while the main difference is in the spin up peak (located at $V \approx 290\mu$V) is significantly less pronounced than the equilibrium model predicts.

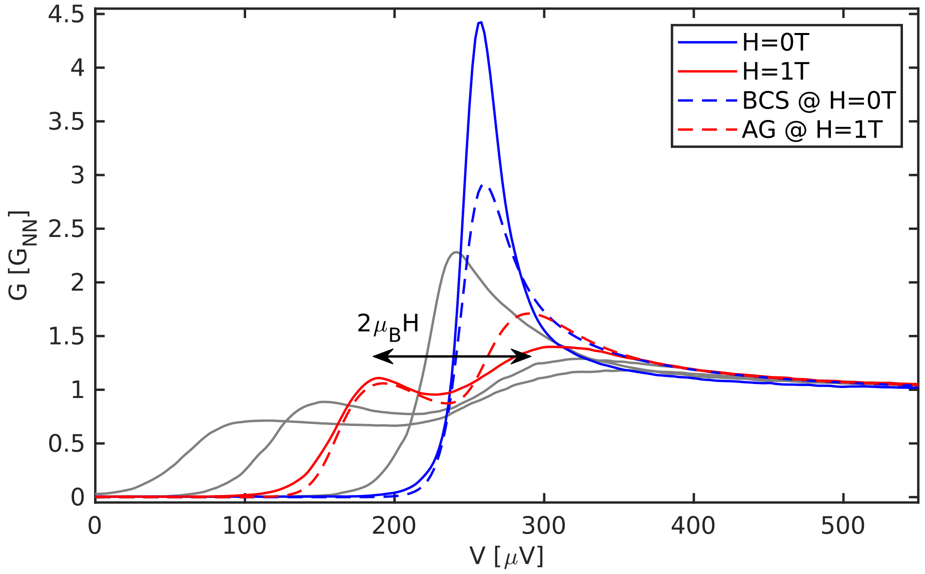

Supplementary Figure 9: The NIS $G(V)$ curves at $H = 0$T (blue) and at $H = 1$T (red). The gray traces are the $G(V)$ traces up to $H \approx 1.8$T in steps of $\Delta H \approx 0.4$T. The dashed blue and red traces are the simulated $G(V)$ curves for a BCS DOS at $T = 90$mK and an Abrikosov-Gor'kov DOS at $H = 1$T and $\alpha = 6.5\mu$eV and the same temperature.

Supplementary figure 10 shows the $G(V)$ of the injection junction J_{inj} as a function of the magnetic field as a color-plot, as well as a numerical simulation of the same using the equilibrium $\Delta(H)$ dependence (based on 23) for comparison.

As a result of the nonequilibrium effects some of the sample properties, in particular the Abrikosov-Gor'kov depairing energy α, cannot be determined from a straightforward fit. The depairing rate was determined to be

$\alpha_0 = 6.5\frac{\mathrm{\mu eV}}{\mathrm{T}^2}$ by using the both the tunneling spectra of the injector junction as well as the detectors which also, albeit indirectly, probe the DOS without significant out-of-equilibrium effects. This value was also used for the theoretical calculation shown in figure 10.

As the AG energy is lower than the Zeeman energy $\alpha(H) = \alpha_0 H^2 < \mu_B H$ all the way up to the critical field, at finite fields the DOS will be well Zeeman split, as shown in the same figure.

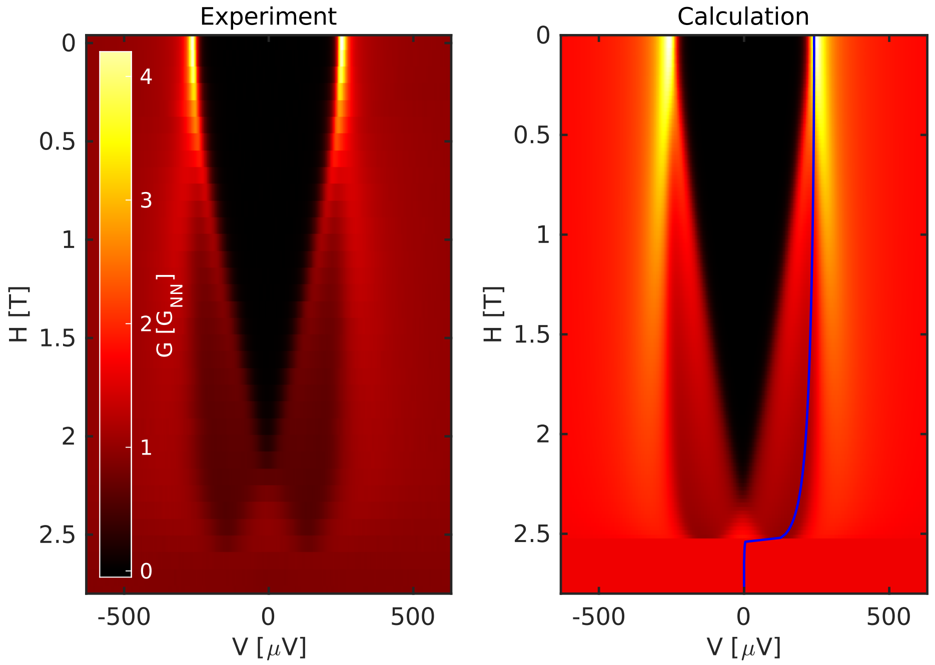

Supplementary Figure 10: A color-map of the NIS $G(V)$ curves as a function of the magnetic field, from the experiment (left), and from the theory using the **equilibrium** self-consistent $\Delta(H)$ (blue curve).

1.2.2 The SIS′ detector

The current across an SIS′ has two main contributions: the Josephson supercurrent and the quasiparticle tunneling current. In this chapter we will discuss how the tunneling current can be used as a spectroscopic probe of the out of equilibrium state in a superconductor, as well as how the supercurrent contribution can be suppressed.

The Josephson current:

The Josephson supercurrent trough an extended SIS junction, at a finite

magnetic field, is given by [26]:

$$I_s = \int_{\frac{-d}{2}}^{\frac{-d}{2}} j_s(x, H) \sin(\varphi_0 + kx) dx \qquad (26)$$

where d is the junction width, and $j_s(x, H)$ describes both the current density profile along the axis orthogonal to the applied magnetic field as well as the field dependence of $\Delta(H)$. The wavenumber $k = 2\pi H(2\lambda + d_{barrier})/\Phi_0$ (λ is the field penetration depth and $\Phi_0 = 2 \times 10^{-15}$Wb the magnetic flux quantum), describes the total magnetic field flux trapped in the junction.

If the barrier is uniform, that is $j_c = const$, the following result is obtained: $I_s(\varphi) = \frac{j_s \sin\left(d\frac{k}{2}\right)}{k/2} \sin(\varphi)$. Then the critical current is always obtained at $\varphi = \frac{\pi}{2}$ and it follows the usual Fraunhofer pattern - it is equal to zero when the trapped flux is equal to one flux quantum.

If, on the other hand, the barrier is not spatially uniform and $j_s(x)$ has an odd component ($x = 0$ is the center of the junction), the supercurrent can be expressed as $I_s(\varphi) = A \sin(\varphi) + B \cos(\varphi)$, where the field dependence is hidden in $A(H)$ and $B(H)$. The critical current then becomes $I_c = \sqrt{A(H)^2 + B(H)^2}$. Because these two coefficients are not simultaneously equal to zero the critical current cannot be fully suppressed by an application of the magnetic field. However the minimum of I_c is still obtained close to the field at which there is one flux quantum in the junction.

Experimentally the critical current can be accessed directly by measuring the $V(I)$ or by measuring the differential conductance $G(V) = \dfrac{\partial I}{\partial V}$ at $V = 0$. Additionally, there can be an excess supercurrent contribution at a finite voltage V, if the the Josephson frequency matches a resonant frequency in the device $2eV = hf$, which for the device in question happens at $V \approx 256\mu$V ($f \approx 124$GHz) - see the inset of figure 11.

Supplementary figure 11 shows the normalized differential conductance at zero voltage bias of detector J_1. As expected from the geometry ($d_{junction} \approx 200$nm, $2\lambda + d_{barrier} \approx 10$nm) the Josephson critical current is minimal around $H = 1$T, and by measuring at (or close to) this magnetic field allows us to probe only the quasiparticle current contribution (as described in the section 1.2.2). The curve does not exactly follow the shape of a (smeared out) cardinal sine function, which is primarily because of the $\Delta(H)$ and $\Delta_D(H)$ dependencies which are not taken into account.

The residual Josephson contribution to the $G(V)$ curve at $H = 1$T, can be modeled as a Gaussian peak and subtracted from the trace, as is shown in figure 16.

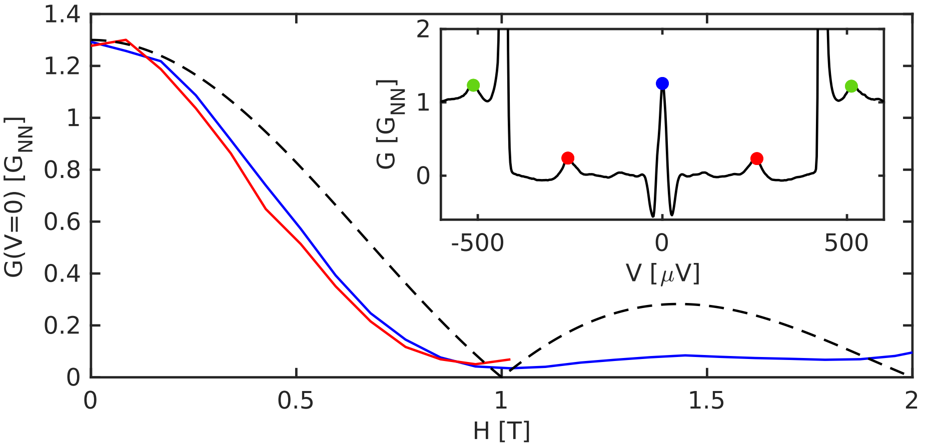

Supplementary Figure 11: The magnetic field dependence of the zero bias Josephson peak ($G(V = 0)$ - blue curve) as well as the first Josephson resonance ($5.5G(V \approx 256\mu V)$ - red curve) for the detector J_1. The red trace stops at $H = 1T$ as slightly above this field the spectral gap closes below the threshold $\Delta + \Delta_D = 256\mu eV$. The black dashed curve shows the Fraunhofer pattern normalized by the I_c at $H = 0$. The inset shows the $G(V)$ curve at zero magnetic field with the Josephson peak, as well as the first two resonances labeled.

The quasiparticle tunneling current:

The tunneling current through an SIS′ junction is (see [21]):

$$I(V) = \frac{1}{eR_{\rm N}} \int_{-\infty}^{\infty} N_D(E + eV) N(E)[f^p(E) - f^p_D(E + eV)]dE \qquad (27)$$

where R_N is the junction resistance, N and N_D are the DOS functions for the probed superconductor and the detector electrode respectively, while f^p and f^p_D are the distribution functions in the particle picture.

By applying a voltage $eV = \Delta + \Delta_D$ to the detector junction the the (electron side) gap edge of the detector is brought down to the gap edge of the superconductor at $E = -\Delta$. As the density of states of the detector is $N_D(E > 2\Delta_D) \approx 1$ and the distribution function $f^p_D(E > 2\Delta_D) = 0$ the electron side of the superconductor is probed by a flat DOS with no excitations present. The tunneling on the hole side is blocked by the spectral gap of the detector down to $E = \Delta - 2\Delta_D$. Therefore the total current will be directly proportional to the number of electron-like excitations in the probed superconductor. Likewise if a negative voltage of the same magnitude is applied the number of hole-like excitations is measured by the tunneling current.

Measuring the $G(V)$ curves can provide spectroscopic information about the quasiparticle population. If the detector is at equilibrium and at a sufficiently low temperature it will host a vanishingly small number of quasiparticles and therefore the term in the $G(V) = \dfrac{\partial I}{\partial V}$ proportional to $N_D(E + eV)\dfrac{\partial f_D^p(E+eV)}{\partial V} \ll 1$ can be neglected ($\frac{\partial f^p}{\partial E}$ is nonzero only in a window of $3.5k_BT \ll \Delta$). The other, nonzero, term is

$$G(V) = \frac{1}{eR_{\rm N}} \int_{-\infty}^{\infty} N(E)[f^p(E) - f_D^p(E+eV)] \frac{\partial N_D(E+eV)}{\partial V} dE$$

At sub-gap voltages ($eV \leq \Delta + \Delta_D$) and in the relevant energy range ($|E| > \Delta$, where $N(E) > 0$), f^p can be replaced with $f^p(E, T = 0)$ (i.e. no quasiparticles are present in the detector which could contribute to the tunneling process), and so the term in the square brackets becomes $\delta f(E) = f^p(E) - f_D^p(E+eV) = f^p(E) - f^p(E, T = 0)$, which just accounts for the (out-of-equilibrium) excitations in the superconductor. Finally the relevant expression for the differential conductance becomes:

$$G(V) = \frac{1}{eR_{\rm N}} \int_{-\infty}^{\infty} \frac{\partial N_D(E+eV)}{\partial V} N(E)\delta f(E) dE \tag{28}$$

The derivative $\frac{\partial N_D(E+eV)}{\partial V}$ is very sharply peaked at $E \approx \Delta_D$, and from this it is clear that the $G(eV = E-\Delta_D) \propto N(E)\delta f(E)$ probes the number of excitations at energy E. This property allows for the use of an SIS junction as a spectroscopic detector of out-of-equilibrium quasiparticles.

A graphical representation showing all of this is given in figure 12, showing the DOS of the probed superconductor, an equilibrium distribution function, an out-of-equilibrium distribution function (only the f_L mode is nonzero), the DOS of the detector as well as it's derivative. Supplementary figure 13 shows the corresponding $I(V)$ and $G(V)$ curves, along with a comparison with $N(E)f(E)$. Although only the electron side of the spectrum is shown in figure 13 the situation is the same on the hole side: $\delta f(E)$ in equation 28 amounts to, at a sufficiently low detector temperature, $\delta f = 1-f$ for $E < 0$. Thus the hole-like excitation density is again probed by $\frac{\partial N_D(E+eV)}{\partial V}$. Additionally, as in our device the gap in the detector is smaller than the gap in the bar, $\Delta_D < \Delta$, for a positive/negative detector voltage the detector hole/electron coherence peak is within the gap of the superconductor, the detector $G(\pm|V|)$ curve probes only the density of electron/hole like excitations at $E = \pm(e|V| + \Delta_D)$. Therefore an even number of hole-like and electron-like excitations will present itself as a detector $G(V)$ even in V, while a charge imbalance (i.e. a

dissimilar number of electrons and holes) will give rise to an odd component of the detector $G(V)$ spectrum.

By integrating the previous expression for the $G(V)$ one again finds that the $I(V)$ measures the number total number of quasiparticles.

The whole discussion holds true in the spin-split case, the only difference is that the different spin channels have to be considered separately and their contributions should be then added, as the spin is conserved by tunneling.

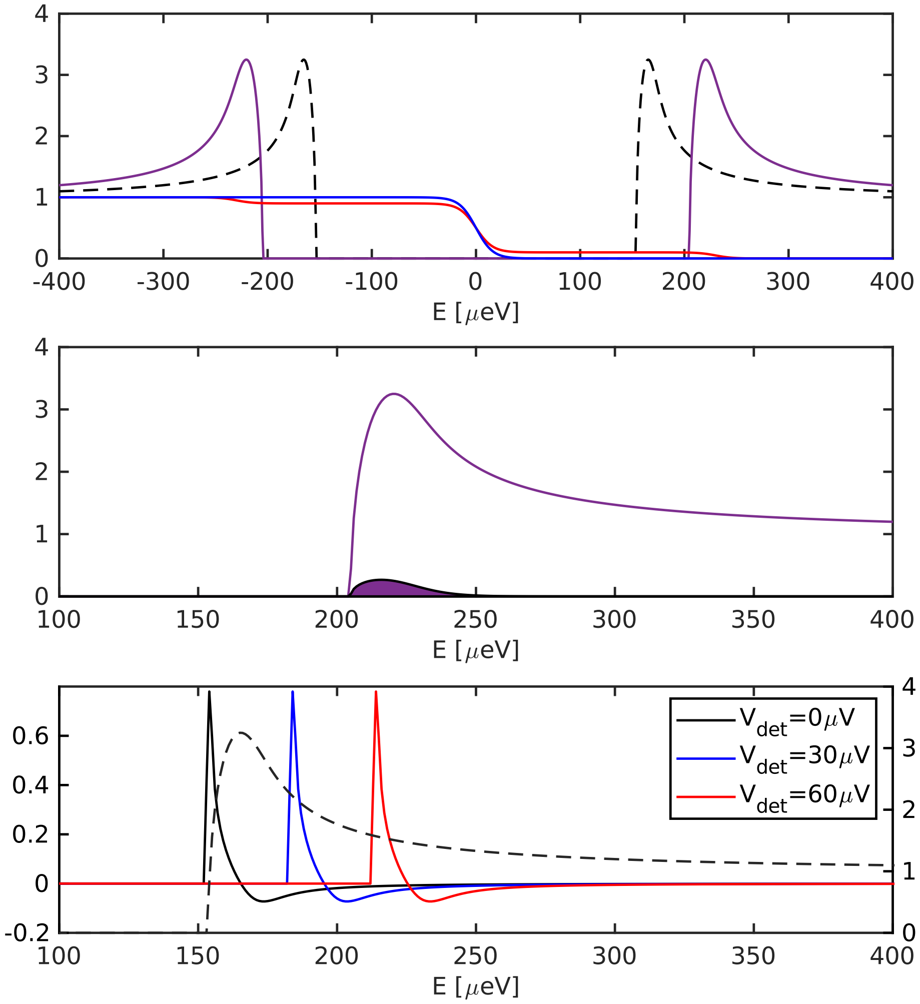

Supplementary Figure 12: Top: the superconductor DOS $N(E)$ (purple), the detector DOS $N_D(E)$ (black dashed), the equilibrium Fermi-Dirac distribution function (blue) and a nonequilibrium one (red, only $f_L \neq 0$). Middle: The density of states as above and the nonequilibrium quasiparticle density $N(E)\delta f(E)$. Bottom: The DOS of the detector (black dashed, right scale), as well as its derivative at $V_{det} = 0\mu V$ (black), $V_{det} = 30\mu V$ (blue) and $V_{det} = 60\mu V$ (red). The two distribution functions shown in the top panel are also used used for the traces in figure 13.

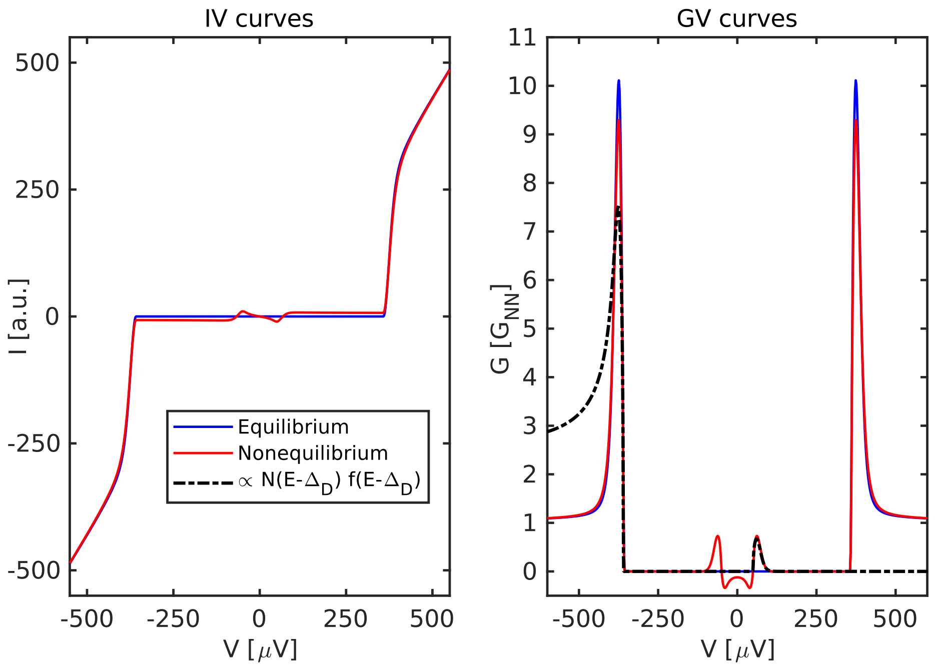

Supplementary Figure 13: Left: the $I(V)$ curve for the equilibrium and non-equilibrium distribution functions shown in figure 12. Right: the corresponding $G(V)$ curves and a comparison with $N(E)f(E)$.

1.2.3 The spin sensitive SIS′ detector

Contrary to an NIS junction, as shown by [25], the $I(V)/G(V)$ traces of an SIS junction will not show the Zeeman splitting under a finite magnetic field. This is because both superconductors become Zeeman split, and as the tunneling process needs to be considered separately for the spin up and spin down, this just amounts to having the *same* shift in the chemical potential/Fermi energy $\pm\mu_B H$ on both sides of the junction. However, if the detector side of the junction is not spin split (i.e. $N_\uparrow \approx N_\downarrow$), which is the case of the spin-mixing induced by spin-orbit interaction in the superconductor [25], it leads to an observable Zeeman splitting in the $G(V)$ curve. The top panel of figure 14 shows the difference between the DOS of a superconductor with a negligible spin-orbit interaction ($R_{\mathrm{SO}} \ll \Delta$, blue traces) and with a large spin-orbit interaction ($R_{\mathrm{SO}} \gg \Delta$, red traces) at $H = 1$T. The bottom panel of the same figure shows the normalized differential conductance of an SIS junction made out of two superconductors with low SO interactions (blue) and an SIS′ where the one has a low and the other a high spin-orbit interaction.

A detector made out of a non-Zeeman-split superconductor can be used as a spin sensitive detector: following the discussion in 1.2.2 the spin down quasiparticles will be detected at a detector voltage of $V_\downarrow = \Delta - \mu_B H - \Delta_D$, while the spin up ones will be detected at $V_\uparrow = \Delta + \mu_B H - \Delta_D$.

Experimentally such a detector can be realized covering the *Al* detector electrode, with a mono-layer of *Pt* [27]. Because of the high atomic number of *Pt* it induces a strong spin-orbit effect in the detector and suppresses the Zeeman splitting. To verify this a separate set of samples, fabricated in roughly the same geometry as the final device, were made with high resistance NIS junctions ($R \approx 250\mathrm{k}\Omega$) to probe the equilibrium DOS. Supplementary figure 15 shows the magnetic field dependence of the DOS, with and without the *Pt* doping, verifying that the doped sample is not Zeeman split. Although such a measurement is not a reliable way to determine the strength of the SO interaction precisely, the key point is that there is a *single* gap edge, which allows the junction to be a spin sensitive spectroscopic detector. The $G(V)$ curve of the SIS′ at a finite magnetic field, showing the Zeeman splitting, is shown in figure 16. For reference the same figure includes a trace from a previous sample at the same field, where the detector electrode was *Al* only and is therefor Zeeman split, and the $G(V)$ traces do not show two separate coherence peaks. The difference in the amplitude of the peaks is due to a lower orbital depairing.

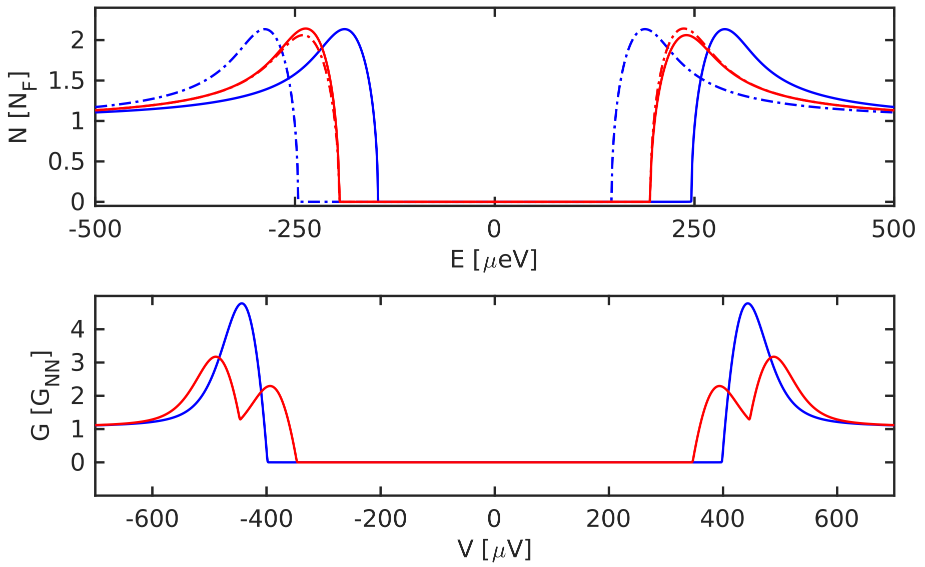

Supplementary Figure 14: Top: The spin up and spin down DOS of a superconductor with $R_{\mathrm{SO}} \ll \Delta$ (blue), and the same for $R_{\mathrm{SO}} \gg \Delta$ (red). Bottom: If the GV curves of two SIS junctions - one couples two Zeeman split superconductors (blue), and the other one couples a Zeeman split superconductor with a non-split one (red).

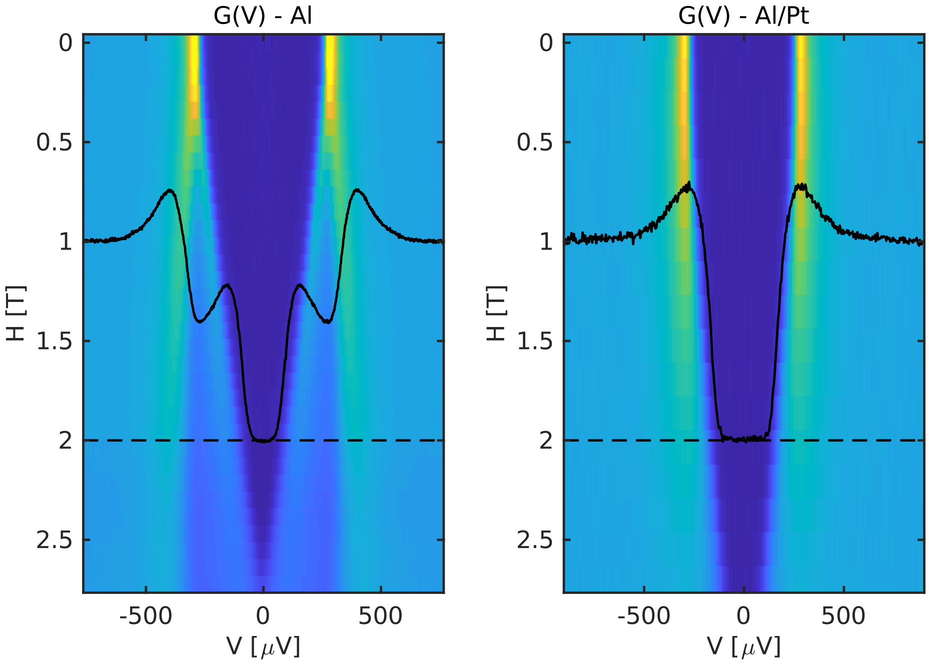

Supplementary Figure 15: The tunneling differential conductance $G(V)$ color-map as a function of the magnetic field for an Al sample (left), and an Al/Pt sample. The black lines are the $G(V)$ traces at $H = 2$T.

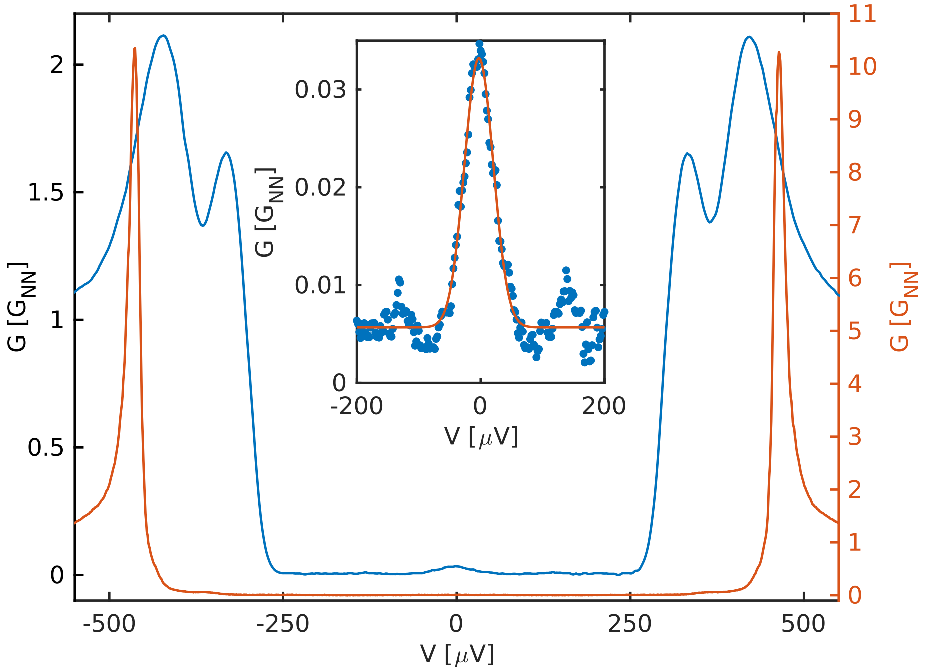

Supplementary Figure 16: The $G(V)$ curve of J_1 at $H = 1$T which shows the "splitting" of the coherence peaks at $|eV_{det}| = |\Delta \pm \mu_B H + \Delta_D|$, which is explained in detail in the section 1.2.3. The small Josephson contribution, close to $V = 0$, can be modeled as a Gaussian peak and subtracted from the data, as shown in the inset. The trace on the right scale is from a previous device with a Zeeman-split detector, also at $H = 1$T.

2 Supplementary Discussion

2.1 Measurement of the number of QP's at high injection

In this chapter the validity of the claim that the quasiparticles relax solely by the thermalization at the ends of the wire is verified. First a theoretical argument is given and than the relevant measurements are presented.

A slight generalization of equations 13 and 16 reads:

$$D^*(E)\nabla^2 f(E) = I_{coll}(f, E) \tag{29}$$

where the $D^*(E)$ is the energy dependent diffusion matrix, $f(E)$ a vector describing the different distribution modes, and $I_{coll}(E)$ takes into account all of the different relaxation and scattering mechanisms.

The charge and spin relaxation processes, as well as an effective electron-electron interaction, conserve the number of quasiparticles. On the other hand the electron-phonon interaction will decrease the QP population through recombination. As a consequence at timescales shorter than the recombination time $\int_{\infty}^{\infty} I_{coll}(f, E)dE = 0$ vanishes for any distribution function f.

If most of the quasiparticles are injected at high energies (i.e. $E \gg \Delta$), where D^* is diagonal and proportional to D_0 (the normal state diffusion coefficient), one can simplify equation 29 by integrating over energy and disregarding the dependence at low energies, one gets:

$$D\nabla^2 f = 0 \tag{30}$$

To obtain a physical solution from the previous equation one must impose the proper boundary conditions, at the injector and at the ends of the wire.

As, at high energies, the number of quasiparticles is proportional only to f as the density of states is constant, according to the equation 30 one should expect a linear spatial dependence/decay of the number of quasiparticles.

As discussed in the section 1.2.2 the $I_D(eV_D = \Delta + \Delta_D)$ probes the total number of quasiparticles in the wire. A measurement of this quantity as a function of the injection current is shown in figure 17, where the data has been re-scaled by the junction resistance and normalized to a unit slope at high injection for J_1. The number of quasiparticles close to the injector (data from the detector J_1) has the following dependence on current: at low injection currents there is a rapid growth of the quasiparticle population, followed by a leveling off in the region $I_{inj} = 10 - 50$nA, after which there is again a linear dependence with a smaller slope than at low injection. The number

of quasiparticles measured by J_2 and J_3 is smaller, in the beginning it has a concave shape and above $I_{inj} = 50$nA it also becomes linear. At high injection currents, when the energy dependence of the diffusion coefficient doesn't play such a big role anymore, as well as when the electron-electron time is finite (resulting in a pseudo-thermal distribution function), the number of quasiparticles is linear with the injection current. The same holds true for the other two detectors J_2 and J_3 (also in figure 17.

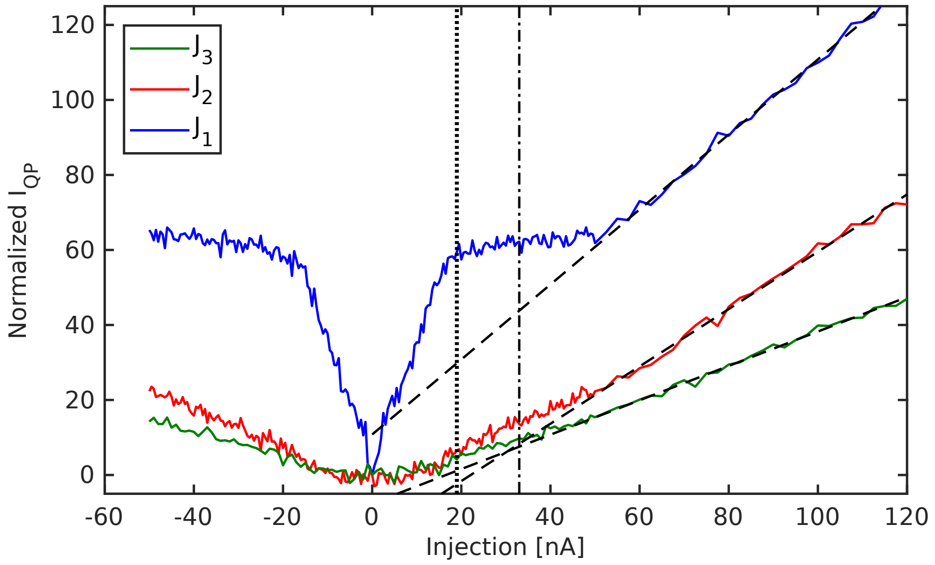

Supplementary Figure 17: The number of quasiparticles measured by each of the detectors at $H = 0$. The data is normalized such that the linear, high injection, part of the J_1 trace have a unit slope. The black dashed lines are linear extrapolations to zero. The dotted vertical line indicates $eV_{inj} \approx 1.33\Delta_0$ and the dash-dot line indicates $eV_{inj} \approx 2\Delta_0$.

In this high injection regime the relation becomes $N_{QP} = k(x)I_{inj}$, where only the slope of the curve depends on the position. If we take the slope itself to be a measure of the number of quasiparticles present in the wire, and plot it versus the position of the detector, we find that it extrapolates to zero at the end of the wire (figure 18). Based on this we can safely argue that the quasiparticles relax to the equilibrium state by thermalization and recombination within the reservoirs at the end of the wire.

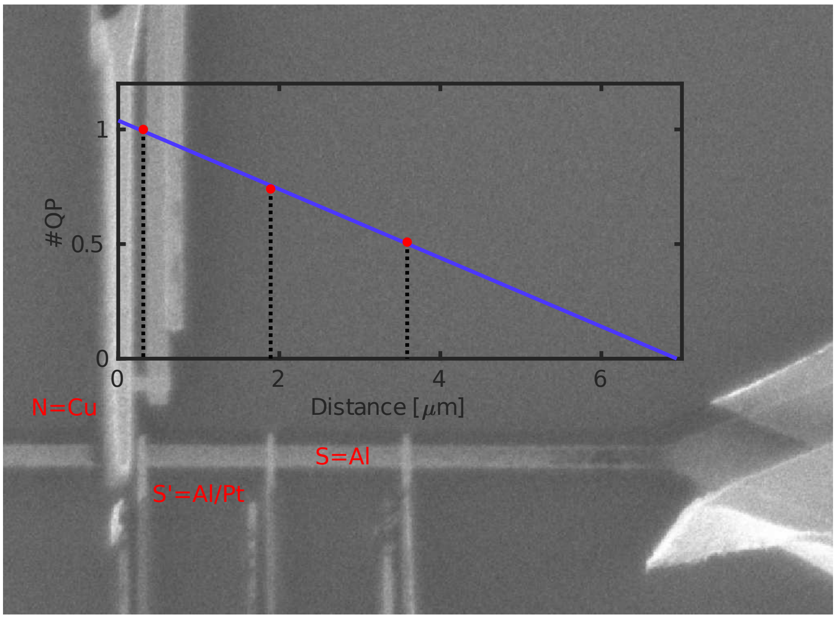

Supplementary Figure 18: An SEM micrograph of the sample showing a wider view, as well as an inlay showing the linear decay of the number of quasiparticles as a function of space and it's extrapolation to zero at the reservoir.

2.2 Nonthermal QP distribution at $H = 1$T

Based on the theory, presented in section 1.1, and as argued in the main text, quasiparticle injection from a normal-metal tunnel junction into a superconductor results in a non-thermal distribution function, characterized by a large number of quasiparticles at the gap, $E = \Delta$, with nonequilibrium quasiparticles up to $E = eV_{inj}$. This is shown at zero magnetic field in figure 3 of the main text: panel c shows the experimental data, while panel d shows the theoretical model. To highlight this step-like feature in the detector $G(V)$ trace the equilibrium $G(V, I_{inj} = 0)$ trace is subtracted from the data (see the black trace in panel b), as the step height is small compared to the nontrivial equilibrium trace (due to the Josephson effect). This allows the step to be observed easily, but it also introduces some artefacts in the data - a purple (no QPs present) region is also found at $V_{det} \approx 120$µeV almost regardless of the injection current. This is precisely the voltage at which a peak appears in the equilibrium $G(V)$ trace (black line in panel b). As quasiparticles are injected the gap, Δ, of the superconductor is reduced, modifying the Josephson coupling, which both reduces the height and slightly moves this background peak.

To show that this nonthermal distribution function persists at higher fields a figure, similar to the one shown in panel c of figure 4 in the main text, is shown here with the NIS trace overlaid on top (see figure 19). At this field ($H = 1$T) the Josephson current is almost completely suppressed, resulting in a trivial background. Additionally, the depairing introduced by the magnetic field smears out both the superconductor and the detector DOS, resulting in a smaller QP peak at $E = \Delta$. Thus it is possible to directly see the same two regions, with QPs below $E = eV_{inj}$ (increased conductance - light blue color), and no QPs above $E = eV_{inj}$ (dark blue color), without subtracting the equilibrium trace. The line separating the two regions is again the NIS $I(V)$ curve (overlaid on top).

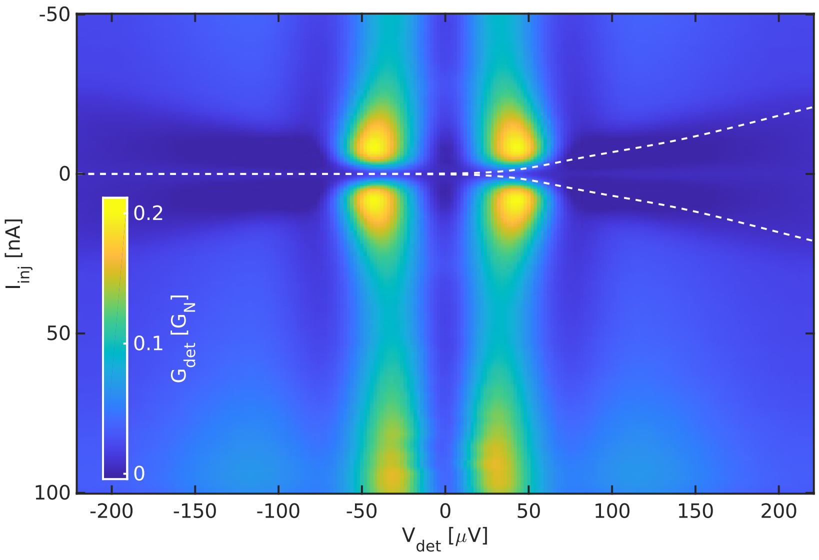

Supplementary Figure 19: The $G(V)$ trace of detector J_1 at $H = 1$T (color-plot), with the injector $I(V)$ curves overlaid on top (dashed white lines). One can identify two regions: below $E = eV_{inj}$ with quasiparticles present (light blue color) and above with no quasiparticles detected (dark blue color).

2.3 Pseudo-thermal QPs far away from the injector

The nonthermal distribution function observed with J_1, presented in the main text at $H = 0$ and at $H = 1$T in 2.2, is in contrast with the behavior observed at the other two detectors J_2 and J_3, shown in figure 20. At these distances the peak at $eV = \Delta - \Delta_D$ is still present but is much less prominent, and at all injection currents which show an increased number of QPs they cannot be confined to a finite energy/voltage range. This is more in line with

a pseudo-thermal distribution function with a long (exponentially decaying) tail, in which a cut-off energy is not well defined.

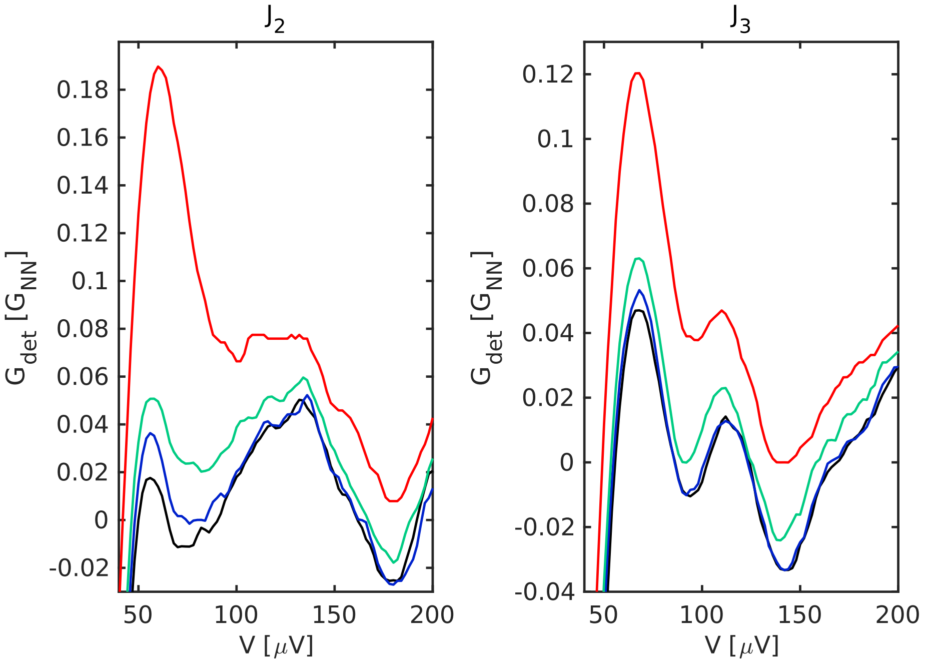

Supplementary Figure 20: The sub-gap $G(V)$ curve of detector J_2 (left) and detector J_3 (right) as a function of the injection current ($H = 0$T). The injection currents are $I_{inj} = 0$nA (black), $I_{inj} \approx 13$nA (blue), $I_{inj} \approx 40$nA (green), $I_{inj} = 120$nA (red.

To show this in a clearer way we compare the number of quasiparticles close to $E = \Delta$ and the total, which is shown in figure 21 for all three detectors. The two traces from J_1 are identically equal up to $I_{inj} \approx 20$nA, which corresponds to $V_{inj} \approx 1.4\Delta_0$, and separate after that. Even at the highest injection shown in the figure more than 75% of the quasiparticles remain in the vicinity of the gap edge. The J_2 and J_3 traces, on the other hand, show a large number of quasiparticles at higher energies, roughly 50% of the total. Again this can be understood in terms of a pseudo-thermal distribution function with a long tail.

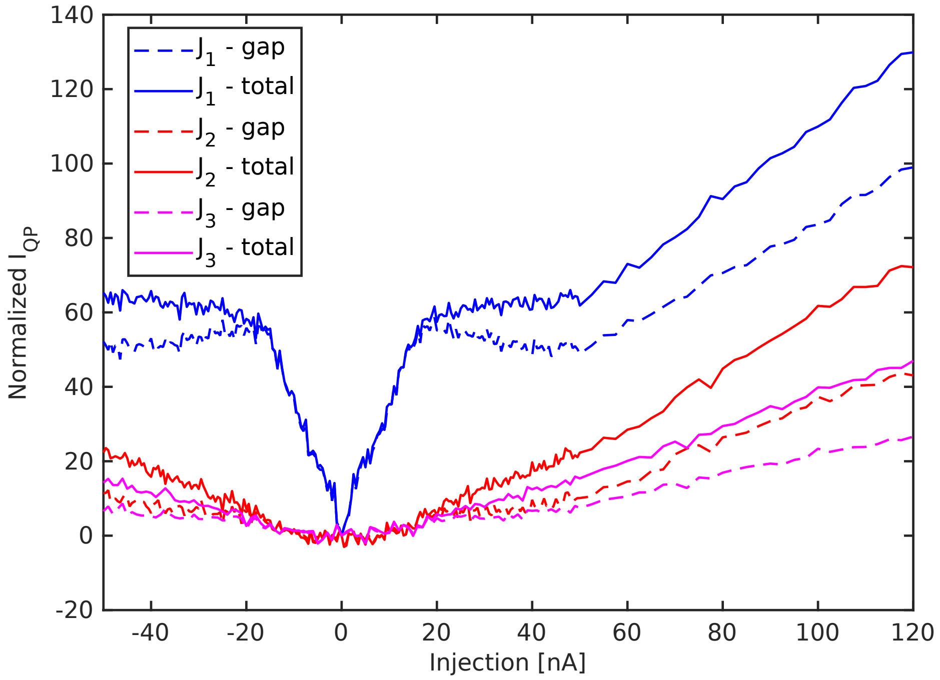

Supplementary Figure 21: The total number of quasiparticles measured with the detectors (full traces) and the number of quasiparticles close to superconducting gap Δ (the QP signal integrated over $40\mu V < V < 100\mu V$, dashed traces) for all three detectors as a function of the injection current. The traces are normalized such that the linear high injection part of the J_1 trace has a slope of unity.

2.4 Spin-resolved QP detection

The same spectroscopic measurements as the ones presented at $H = 0$ and $H = 1$T in the main text can be performed at other magnetic fields. The fundamental difference between $H = 0$ and finite fields is that the distribution functions for the two spin species will not necessarily be the same: when the injector is biased such that $\Delta - \mu_B H < V_{inj} < \Delta + \mu_B H$ the DOS of the superconductor behaves as an almost perfect spin filter and quasiparticles of one spin species are preferentially injected/excited. On top of this, as the detector electrode is not spin split (due to the Pt monolayer on top) one should expect to see two peaks in the detector $G(V)$, the first at $eV = \Delta - \mu_B H - \Delta_D$ and the other one at $eV = \Delta + \mu_B H - \Delta_D$ which probe the two spin states separately, allowing for a spin sensitive spectroscopic study without the need for a spin polarized barrier. Supplementary figure 22 shows the $G(V)$ curve of the detector for $I_{inj} = 40$nA and $I_{inj} = 5$nA,

which corresponds to the maximum spin polarized current at higher fields, as a function of the applied magnetic field. For clarity the traces are offset such that $eV_{det} = \Delta - \Delta_D$ is at $V_{det} = 0$. At the higher of the two currents, when both spin up and spin down electrons are injected, two peaks are visible, separated by $2\mu_B H$ as indicated by the vertical black lines.

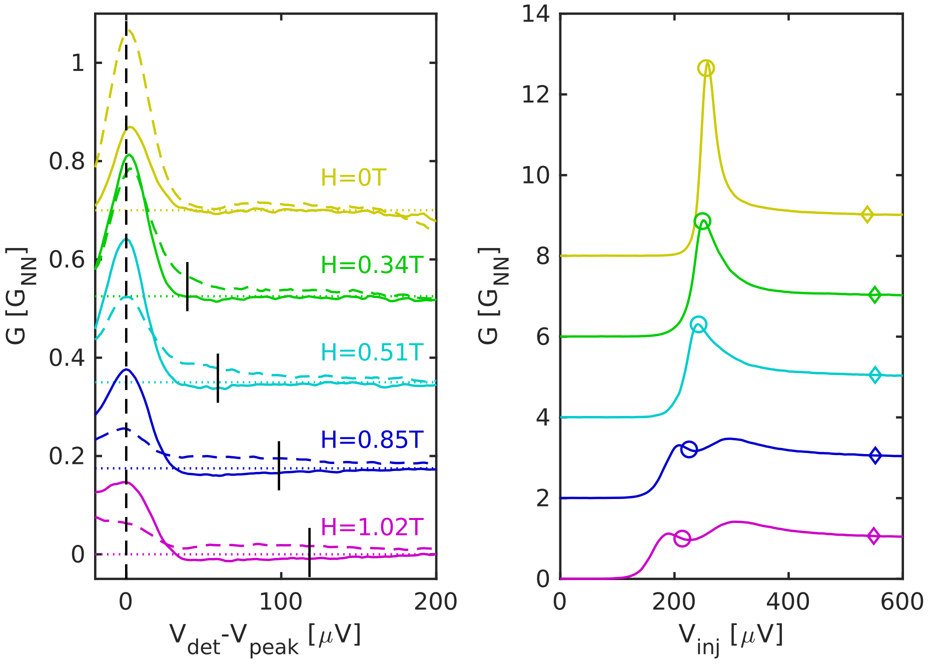

Supplementary Figure 22: The detector $G(V)$ at $I_{inj} = 40\text{nA}$ and $I_{inj} = 5\text{nA}$ for different fields, showing the detection of both spin down and spin up quasiparticles. The traces are offset such that the (dominant) spin down peak is at $V_{det} = 0$ and the $G(V)$ at $I_{inj} = 0$ is subtracted. The right panel shows the injector $G(V)$ curves at the same field as on the left. The circle and the diamond show the injection voltage at which $I_{inj}(V_{inj}) = 5\text{nA}$ and $I_{inj}(V_{inj}) = 40\text{nA}$ respectively.

2.5 Differentiating between the f_T and the f_{L3} modes

As both of these modes contribute to the charge imbalance in a superconductor one cannot distinguish between them by just measuring the voltage across a nearby normal metal probe, as was done previously [20]. The distinction between the two can, however, be made by performing a spectroscopic measurement: as shown in the section 1.1.4 the f_T mode is dominantly present at low magnetic fields and at high injection energies, while the spin-energy

mode f_{L3} is localized within $\Delta - \mu_B H < E < \Delta + \mu_B H$ and becomes more visible with increasing filed, but dies off close to H_c.

To verify that the odd signal shown in the main text is indeed due to the spin-energy the following analysis was performed. The left panel of figure 23 shows the $G(V)$ of detector J_1 at $H = 0$T for $I_{inj} = \pm 120$nA (the figure is presented in a similar fashion as in 22, offset such that the QP peak is at zero and the equilibrium trace is subtracted). Above the QP peak there is a difference between the traces, which changes sign with the sign of the injection current and detection voltage (only the positive part of the trace is shown here for clarity), indicating the presence of a charge imbalance induced by the f_T mode ($f_{\mathrm{L3}} = 0$ at $H = 0$ in our experiment). The odd component of the trace can be integrated over the regions marked in the figure (the negative peak at $\approx 200\mu$V is related to the Josephson current and is therefore omitted), and the result is shown on the right panel of figure 23. The same panel also shows the results of a similar procedure done for $H = 1$T (integrated in the region $90\mu\mathrm{V} \leq V_{det} \leq 210\mu\mathrm{V}$), which shows no asymmetry, signifying that the contribution of the f_T mode at $H = 1$T is negligible.

Together with the results shown in the main text, this shows that the charge imbalance observed at finite fields and close to the gap edge of the superconductor is associated with the presence of the f_{L3} mode excited by quasiparticle injection into a Zeeman split superconductor through an NIS junction.

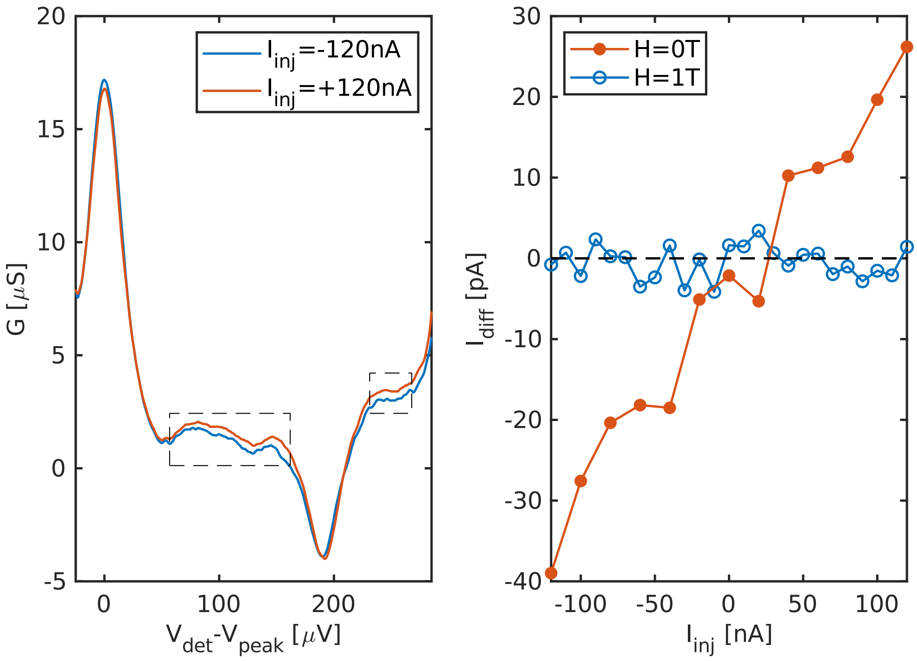

Supplementary Figure 23: A set of $G(V)$ curves from the detector J_1 at $H = 0$T. For each of the injection currents there are two traces: one for a nominally positive current (red) and the other for the negative current (blue). An odd component, corresponding to a charge imbalance can only be observed above $I \approx 40$nA and starting from high energies, in accordance with the properties for the f_T mode.

Supplementary References

[1] Nikolai Kopnin. *Theory of nonequilibrium superconductivity*. Vol. 110. Oxford University Press, 2001. DOI: 10.1093/acprof:oso/9780198507888.001.0001.

[2] EM Lifshitz and LP Pitaevskii. "Statistical Physics (Part 2), Vol. 9 of Landau and Lifshitz". In: *Course of Theoretical Physics, 2nd ed.(Pergamon, Oxford, 1980)* (1980).

[3] Aleksei Alekseevich Abrikosov, Lev Petrovich Gor'kov, and Igor' Ekhiel'evich Dzyaloshinskii. *Quantum field theoretical methods in statistical physics*. Vol. 4. Pergamon, 1965.

[4] Richard D Mattuck. *A guide to Feynman diagrams in the many-body problem*. Courier Corporation, 1992.

[5] Piers Coleman. *Introduction to many-body physics*. Cambridge University Press, 2015.

[6] Gert Eilenberger. "Transformation of Gor'kov's equation for type II superconductors into transport-like equations". In: *Zeitschrift für Physik A Hadrons and nuclei* 214.2 (1968), pp. 195–213. DOI: 10.1007/BF01379803.

[7] Klaus D Usadel. "Generalized diffusion equation for superconducting alloys". In: *Physical Review Letters* 25.8 (1970), p. 507. DOI: 10.1103/PhysRevLett.25.507.

[8] Mihail Silaev et al. "Long-range spin accumulation from heat injection in mesoscopic superconductors with zeeman splitting". In: *Physical review letters* 114.16 (2015), p. 167002. DOI: 10.1103/PhysRevLett.114.167002.

[9] F Sebastian Bergeret et al. "Colloquium: Nonequilibrium effects in superconductors with a spin-splitting field". In: *Reviews of Modern Physics* 90.4 (2018), p. 041001. DOI: 10.1103/RevModPhys.90.041001.

[10] Tero T. Heikkilä et al. "Thermal, electric and spin transport in superconductor / ferromagnetic-insulator structures". In: *Progress in Surface Science* 94.3 (2019), p. 100540. ISSN: 0079-6816. DOI: https://doi.org/10.1016/j.progsurf.2019.100540.

[11] Jan Petter Morten, Arne Brataas, and Wolfgang Belzig. "Spin transport in diffusive superconductors". In: *Phys. Rev. B* 70 (21 2004), p. 212508. DOI: 10.1103/PhysRevB.70.212508.

[12] M. J.D. Powell. "A Fortran subroutine for solving systems of nonlinear algebraic equations". In: (Nov. 1968). URL: https://www.osti.gov/biblio/4772677-fortran-subroutine-solving-systems-nonlinear-algebraic-equations.

[13] D. Esteve F. Pierre H. Pothier and M.H. Devoret. "Energy Redistribution Between Quasiparticles in Mesoscopic Silver Wires". In: *Journal of Low Temperature Physics* 118.5 (2000), pp. 437–445. ISSN: 1573-7357. DOI: 10.1023/A:1004606420464.

[14] Kei Yosida. "Paramagnetic susceptibility in superconductors". In: *Physical Review* 110.3 (1958), p. 769. DOI: 10.1103/PhysRev.110.769.

[15] John Clarke. "Experimental observation of pair-quasiparticle potential difference in nonequilibrium superconductors". In: *Physical Review Letters* 28.21 (1972), p. 1363. DOI: 10.1103/PhysRevLett.28.1363.

[16] Thomas R. Lemberger. "One-to-one correspondence of charge-imbalance relaxing mechanisms with pair-breaking mechanisms in superconductors". In: *Phys. Rev. B* 29 (9 1984), pp. 4946–4950. DOI: 10.1103/PhysRevB.29.4946.

[17] Yositake Takane and Yasushi Nagato. "Magnetic Field Effect on Charge Imbalance Conversion in Superconducting Wires". In: *Journal of the Physical Society of Japan* 77.9 (2008), p. 093713. DOI: 10.1143/JPSJ.77.093713.

[18] R Meservey and PM Tedrow. "Properties of very thin aluminum films". In: *Journal of Applied Physics* 42.1 (1971), pp. 51–53. DOI: 10.1063/1.1659648.

[19] SB Kaplan et al. "Quasiparticle and phonon lifetimes in superconductors". In: *Physical Review B* 14.11 (1976), p. 4854. DOI: 10.1103/PhysRevB.14.4854.

[20] C. H. L. Quay et al. "Spin imbalance and spin-charge separation in a mesoscopic superconductor". In: *Nature Physics* 9.2 (2013), pp. 84–88. ISSN: 1745-2481. DOI: 10.1038/nphys2518.

[21] Michael Tinkham. *Introduction to superconductivity*. Courier Corporation, 2004.

[22] Peter Fulde. "High field superconductivity in thin films". In: *Advances in Physics* 22.6 (1973), pp. 667–719. DOI: 10.1080/00018737300101369.

[23] Ro A Klemm, A Luther, and MR Beasley. "Theory of the upper critical field in layered superconductors". In: *Physical Review B* 12.3 (1975), p. 877. DOI: 10.1103/PhysRevB.12.877.

[24] R Meservey, PM Tedrow, and Peter Fulde. "Magnetic field splitting of the quasiparticle states in superconducting aluminum films". In: *Physical Review Letters* 25.18 (1970), p. 1270. DOI: 10.1103/PhysRevLett.25.1270.

[25] R. Meservey, P. M. Tedrow, and Ronald C. Bruno. "Tunneling measurements on spin-paired superconductors with spin-orbit scattering". In: *Phys. Rev. B* 11 (11 1975), pp. 4224–4235. DOI: 10.1103/PhysRevB.11.4224.

[26] Gianfranco Paterno Antonio Barone. ""Small" Junctions in a Magnetic Field". In: *Physics and Applications of the Josephson Effect*. John Wiley and Sons, Ltd, 2005. Chap. 4, pp. 69–95. ISBN: 9783527602780. DOI: 10.1002/352760278X.ch4.

[27] P. M. Tedrow and R. Meservey. "Critical magnetic field of very thin superconducting aluminum films". In: *Phys. Rev. B* 25 (1 1982), pp. 171–178. DOI: 10.1103/PhysRevB.25.171.
